# Evaluation of the Cytotoxic, Antioxidative and Antimicrobial Effects of *Dracocephalum moldavica* L. Cultivars

**DOI:** 10.3390/molecules28041604

**Published:** 2023-02-07

**Authors:** Ștefania Simea, Irina Ielciu, Daniela Hanganu, Mihaela Niculae, Emoke Pall, Ramona Flavia Burtescu, Neli-Kinga Olah, Mihai Cenariu, Ilioara Oniga, Daniela Benedec, Marcel Duda

**Affiliations:** 1 Department of Crop Science, Faculty of Agriculture, University of Agricultural Sciences and Veterinary Medicine Cluj-Napoca, 400372 Cluj-Napoca, Romania; 2 Department of Pharmaceutical Botany, Faculty of Pharmacy, “Iuliu Haţieganu” University of Medicine and Pharmacy Cluj-Napoca, 400010 Cluj-Napoca, Romania; 3 Department of Pharmacognosy, Faculty of Pharmacy, “Iuliu Haţieganu” University of Medicine and Pharmacy Cluj-Napoca, 400000 Cluj-Napoca, Romania; 4 Department of Clinical Sciences, University of Agricultural Sciences and Veterinary Medicine Cluj-Napoca, 400374 Cluj-Napoca, Romania; 5 SC PlantExtrakt SRL, 407059 Rădaia, Cluj-Napoca, Romania; 6 Department of Therapeutical Chemistry, Pharmaceutical Industry and Biotechnologies, Faculty of Pharmacy, “Vasile Goldiș” Western University from Arad, 310048 Arad, Romania

**Keywords:** *D. moldavica*, cultivars, extract, polyphenols, cytotoxic, antioxidant, antimicrobial, BJ cell lines, DLD-1 cell lines

## Abstract

The aim of the present study was to correlate the antioxidant, antimicrobial, and cytotoxic activities of hydroalcoholic extracts obtained from the aerial parts of three *Dracocephalum moldavica* L. cultivars with their polyphenolic compositions. The polyphenols were identified and quantified using spectrophotometrical methods and LC–MS analysis. Their antioxidant capacities were assessed using the 1,1-diphenyl-2-picrylhydrazyl (DPPH) and ferric reducing antioxidant power (FRAP) methods. Their in vitro antimicrobial efficacies were assessed using the agar well diffusion and broth microdilution methods. Their cytotoxicity was investigated on normal diploid foreskin fibroblasts (BJ) and on colorectal adenocarcinoma (DLD-1) cell lines. The results pointed out significant amounts of polyphenolic compounds in the compositions of the tested cultivars, with rosmarinic acid as the main compound (amounts ranging between 5.337 ± 0.0411 and 6.320 ± 0.0535 mg/mL). All three cultivars displayed significant antioxidant (IC_50_ ranging between 35.542 ± 0.043 and 40.901 ± 0.161 µg/mL for the DPPH assay, and for the FRAP assay 293.194 ± 0.213 and 330.165 ± 0.754 µmol Trolox equivalent/mg dry vegetal material) and antimicrobial potential (especially towards the Gram-positive bacteria), as well as a selective toxicity towards the tumoral line. A significant positive correlation was found between antioxidant activity and the total phenolic acids (*r*^2^ = 0.987) and polyphenols (*r*^2^ = 0.951). These findings bring further arguments for strongly considering *D. moldavica* cultivars as promising vegetal products, which warrants further investigation.

## 1. Introduction

*Dracocephalum moldavica* L., traditionally known as the Moldavian balm or Moldavian dragonhead, is a species belonging to the Lamiaceae family, widely known for its aromatic properties [1,2]. It is an annual herbaceous species that can be found in temperate areas, being native to Central Asia and naturalized in Europe [2]. In Romania, the species is rarely found in spontaneous flora, and is only found in limited areas of the eastern, northwestern, and southern parts [3]. The species has thin, brown roots; petiolated lower leaves; crenate–serrate margins; and sessile upper leaves with spiny-serrate edges and is straight, reddish, and strongly branched at the base stem. The corolla of flowers is usually purple–blue and, less frequently, light blue or white, and the fruits are brown, ovoid, and tetranucleate [3,4]. Its aerial parts are traditionally used for the treatment of stomach and liver pathologies, headaches, snake bites, stomatitis, or fungal infections [2,5,6,7]. Essential oils found in the vegetal medicinal product have a citruslike flavor and are rich in oxygenated acyclic monoterpenes, such as geranial, neral, and geranyl acetate [1,2,8,9,10,11]; however, the species is also known for its polyphenolic composition, among which phenolic acids (rosmarinic and caffeic acids) and flavonoids (apigenin, luteolin, and kaempferol) are the main compounds [6,11,12,13,14]. Among flavonoids, tilianin is an interesting glycoside with important biological properties that was firstly isolated from this species [15,16,17]. Both classes of compounds are correlated to the biological activities of the species, such as its antioxidant [1,6,11,12,13,18,19,20], antimicrobial [1,18,21,22], and cardioprotective [15,23,24,25,26,27] effects, which are the most studied ones, together with its anti-inflammatory [28,29], neuroprotective [16,30], sedative [31], cytotoxic [14,32], and antidepressant [33] effects, which are lesser known. The antimicrobial potential of the species was attributed to its essential oils, linked to its antioxidant capacity and demonstrated against *Staphylococcus aureus*, *Escherichia coli*, *Salmonella Typhimurium*, and *Listeria monocytogenes* [1,18]. A hydroalcoholic extract obtained from the aerial parts of the species produced significant results against even antibiotic-resistant strains of *E. coli* and *Klebsiella pneumoniae* [21] and *Staphylococcus aureus* [22]. On the other hand, the cytotoxic activity of the species is less documented in previously performed studies, being attributed to flavonoids and studied on multiple myeloma and acute myeloid leukemia cell lines [14], but also to triterpenes and tested against cervical, lung, and hepatic cancerous cell lines [32]. The vast majority of the biological activities of this species are strongly related to its antioxidative mechanisms [16,34]. The antioxidant potential of the species is its most studied biological activity and is most frequently linked to the flavonoids composition [6,12,13,20], but also to its essential oils [11,18,19]. It is tested especially in vitro by using DPPH [6,11,12,18], 2,20-azinobis (3-ethylbenzothiazoline-6-sulfonate) (ABTS) [12,13,18], superoxide anion [12,13], and iron reduction and chelation [13] scavenging activity assays, but also enzymatic assays such as peroxidase, guaiacol peroxidase, and superoxide dismutase ones [19].

Nowadays, an increasing trend toward the consumption of medicinal and aromatic plants from crops is found worldwide [35,36]. There is a growing demand for medicinal plants to produce herbal medicines, health products, dietary supplements, or cosmetics. The vegetal raw material from medicinal plant crops is insufficient to cover the needs of this industry. There is a preference for cultivated vegetal material because most pharmaceutical companies prefer the raw material that meets the requested quality conditions. Expanding the cultivation of medicinal species and introducing new ones into cultures becomes therefore an important objective [37,38]. All these are complemented by other advantages of cultivation (e.g., modern mechanized agricultural techniques, irrigation, fertilization, and mechanized harvesting) [37].

The cultivation of medicinal species is an important practice worldwide, as the chemical compositions of medicinal species may vary depending on their geographical locations, climates, and cultivars, influencing their biological activities [3,6,9,39]. Although genetically controlled, the biosynthesis of secondary metabolites is strongly influenced by not only environmental and ecological conditions but also agronomic conditions, such as harvesting and processing [2,40]. It is essential to harvest medicinal species when they reach their maximum concentration of active compounds, therefore assuring a raw vegetal product with a high quality [2].

Among the species that are given special importance in agricultural systems, economic development, and health-related aspects, *D. moldavica* is one of the most frequently found species [41,42]. In the case of this species, the need to introduce it into cultures is a consequence of the fact that this valuable medicinal plant with specific active principles is rare in spontaneous flora.

In this context, the aim of the present study was represented by evaluating the chemical profiles and cytotoxic and antimicrobial potentials of three *D. moldavica* cultivars, in relation with their antioxidative capacities. Assessments of their cytotoxic and antibacterial activities linked to their antioxidative mechanisms are poorly studied in the scientific literature [6,13]. Taking all this into consideration, the present study brings novelty and originality by aiming to connect these biological activities with a less-studied class of compounds in the composition of the species, polyphenols. Moreover, the present study aims to provide further arguments to establish the proper cultivation conditions for the species and the right cultivar for further studies on the medicinal potential of its vegetal product.

## 2. Results

Three different *D. moldavica* cultivars (samples A1, A2, and B2) were subjected to phytochemical analysis and antioxidant, antimicrobial, and cytotoxic potential evaluation. This assessment aimed primarily to characterize their chemical compositions and selected biological activities, but also to establish cultivar-related particularities and possible connections with their polyphenolic profiles.

### 2.1. Total Polyphenolic, Flavonoid, and Phenolic Acid Content

Table 1 contains the results of the assessment of total polyphenolic content (TPC), flavonoid content (TFC), and phenolic acid content (TPAC), obtained using spectrophotometrical methods.

Differences were noticed when comparing the amounts of total flavonoid and phenolic acid in *D. moldavica* samples. The most significant variation (*p* < 0.001) was found for TPAC, with its most elevated level in the B2 cultivar. In fact, the B2 sample presented significantly higher amounts of TPC compared to A1 (*p* < 0.05) and A2 (*p* < 0.001).

### 2.2. LC–MS Analysis

The results obtained using LC–MS identification and quantification of the individual components of the three *D. moldavica* samples can be found in Table 2 and Figures S1–S17.

It can be clearly observed that, in the compositions of all tested samples, rosmarinic acid was the main compound, its highest amount being found in the composition of the B2 cultivar. For the A1 sample, apigenin was the compound found with the second highest amount, followed by luteolin-7-*O*-glucoside, ellagic acid, and caffeic acid. For the A2 sample, luteolin-7-*O*-glucoside was found with the highest amount after rosmarinic acid, followed by apigenin, caffeic acid, hyperoside, and chlorogenic acid. Regarding the B2 sample, as in the case of the A1 sample, apigenin was the compound found with the second highest amount, followed by luteolin-7-*O*-glucoside, caffeic acid, and chlorogenic acid. It is therefore clearly noticed that flavonoids and phenolic acids are the main classes of compounds in the composition of the species.

### 2.3. Antioxidant Capacity Assays

An assessment of antioxidant capacity was performed using the DPPH bleaching assay and the FRAP method. Results can be found in Table 3.

In terms of antioxidant activity, the most efficient sample was also found to be the B2 one. Its potential was significantly higher (*p* < 0.001) compared to that of A1 as indicated by the DPPH method (35.650 ± 0.063 µg/mL) and FRAP assay (330.165 ± 0.754 µmol Trolox equivalent/g dry plant material). In addition, a valuable ability to reduce ferric ions was also noticed for sample A2 (301.493 ± 0.115 µmol Trolox equivalent/g dry plant material).

### 2.4. Antibacterial Assays

Results of the in vitro antimicrobial potential evaluation are presented in Table 4 (inhibition zones) and Table 5 (MIC index).

All three *D. moldavica* samples manifested in vitro antimicrobial potential, with significant variations depending on the bacterial species. The highest intensity of antibacterial activity was noticed against the two Gram-positive bacteria (MSSA > MRSA). The sample B2-derived extract was proven to have the most elevated ability to inhibit the bacterial growth of *Staphylococcus aureus* strains (MSSA and MRSA) with diameter zones of 20.50 ± 0.55 mm and 23.50 ± 0.55 mm, respectively. Additionally, the extracts obtained from the A1 and A2 samples induced inhibition zones similar to those of one of the positive controls, gentamicin (*p* > 0.05), but significantly lower (*p* < 0.05) compared to the B2 extract. However, compared to those of amoxicillin–clavulanic acid, these diameters were significantly smaller (*p* < 0.05). As for the Gram-negative bacteria, no inhibitory effects were recorded towards the *Pseudomonas aeruginosa* reference strain, while *Escherichia coli* showed in vitro susceptibility especially towards the sample B2 extract.

The established MIC and MBC values, collected using a broth microdilution method, (Table 5) further confirmed the superior efficacy of B2 sample-derived extract and also indicated the bactericidal effects exhibited by all three extracts according to the resulting MIC index (MBC/MIC ≤ 4).

### 2.5. Cytotoxicity Evaluation

**Figure 1:**
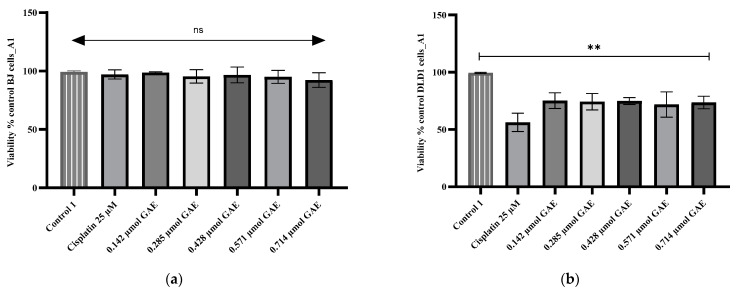
Viability percentages obtained for (**a**) BJ and (**b**) DLD-1 cells after 24 h incubation with A1 extract. The A1 concentrations were calculated according to the TPC µmol GAE (0.142–0.714 µmol GAE). Control 1 (negative control)—untreated cells; control 2—cells treated with cisplatin (25 µM). Data represent the mean ± SD of three independent experiments; ns—no significant differences; **—*p* < 0.05.

**Figure 2:**
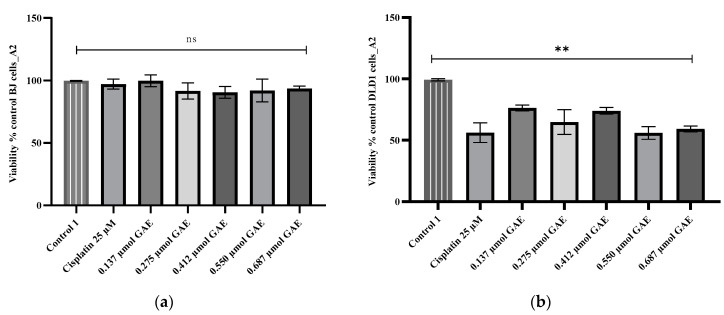
Viability percentages obtained for (**a**) BJ and (**b**) DLD-1 cells after 24 h incubation with A2 extract. The A2 concentrations were calculated according to the TPC µmol GAE (0.137–0.687 µmol GAE). Control 1 (negative control)—untreated cells; control 2—cells treated with cisplatin (25 µM). Data represent the mean ± SD of three independent experiments; ns—no significant differences; **—*p* < 0.05.

**Figure 3:**
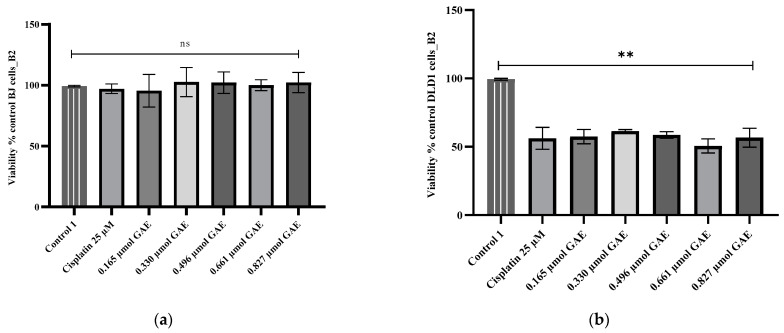
Viability percentages obtained for (**a**) BJ and (**b**) DLD-1 cells after 24 h incubation with B2 extract. The B2 concentrations were calculated according to the TPC µmol GAE (0.165–0.827 µmol GAE). Control 1 (negative control)—untreated cells; control 2—cells treated with cisplatin (25 µM). Data represent the mean ± SD of three independent experiments; ns—no significant differences; **—*p* < 0.05.

The CCK-8 assay was performed to determine the cytotoxicity potential of the *D. moldavica* samples on BJ and DLD-1 cells. As is shown in Figure 1a, Figure 2a and Figure 3a, the evaluated samples (A1, A2, and B2) and cisplatin showed no significant effect (*p* > 0.05) on BJ cell survival. In DLD-1 cells, the tested samples led to a significant decrease (*p* < 0.05) in cell proliferation (Figure 1b, Figure 2b and Figure 3b). The A1 extract led to cell viability percentages ranging between 71.80% ± 5.51 and 75.16% ± 3.44, as induced by concentrations of 0.571 µmol GAE and 0.142 µmol GAE, respectively. Thus, the cytotoxic potential determined for all A1 extract concentrations was significant (*p* < 0.05 compared to the negative control) and still significantly lower (*p* < 0.05) than the positive control, represented by cisplatin-treated cells (56.19 ± 4.02), with IC_50_ = 0.466 µmol GAE (Figure 1b).

This pattern of cytotoxicity was also noticed for the A2 extract, but only for its concentrations ranging between 0.137 and 0.412 µmol GAE, while concentrations of 0.550 and 0.687 µmol GAE (IC_50_ = 0.40 µmol GAE) exhibited cytotoxic effects similar to those of the positive control, cisplatin (*p* > 0.05) (Figure 2b). The most intense cytotoxicity was recorded for extract B2, where the average cell viability was 56.83% ± 3.58 (*p* < 0.05 compared to the negative control), with an IC_50_ of 0.54 µmol GAE. No significant differences were found between the viability percentages calculated for any of the tested concentrations and cisplatin (*p* > 0.05) (Figure 3b). The decrease in cell viability is correlated with the concentration of TPC mg/g GAE.

## 3. Discussion

Medicinal plants have a long history of use for the treatment of a large variety of ailments, including infectious or cancerous disorders, and there are a large number of species that have been studied for their potential therapeutic properties [34]. At the basis of numerous of these ailments, antioxidative mechanisms exhibit one of the most important roles [43]. Natural compounds such as essential oils [44] or flavonoids [45], through their natural ability to scavenge reactive oxygen species (ROS), are among the most studied compounds for antimicrobial or cytotoxic activities because they have a core antioxidative mechanism. In this context, the present study brings novelty and originality by aiming to provide scientific arguments for the cytotoxic and antibacterial activities of *D. moldavica* polyphenols, exhibited using antioxidant mechanisms, as well as for the benefits of its cultivation.

Among the total content of polyphenols, flavonoids, and phenolic acids in *D. moldavica* compositions, TPC and TFC have been previously measured, but in lower amounts than in the present study [12,13]. Their amounts were significantly increased after special treatments with abscisic acid and increased temperature [46], abscisic acid and different watering conditions [47,48], and different ammonium to nitrate ratios [49]; however, even using these methods, their amounts proved to be lower than the ones identified in this study. Only salinity proved to enhance TPC and TFC in higher amounts [50]. Other reports show that amounts of TPC and TFC may also depend on the solvent used for extraction, with ethyl acetate being the most effective [11,24], followed by methanol [6,11]. Ethanol proved to be effective only in the enhancement of TFC [51]. However, amounts that were quantified were inferior to the ones obtained in the present study, bringing originality to this study. The novelty of the present study consists in the quantification of TPAC, which, to the best of our knowledge, has not been previously reported in the composition of the species. TFC was correlated to anti-atherosclerosis activity [52], while TFC and TPC were found responsible for antioxidative effects [6,27,50]. As for external cultivation conditions, abscisic acid treatment and temperature determined the significant increase in TFC and TPAC contents [46].

The chemical compositions of the three cultivars were dominated by the presence of flavonoids and phenolic acids. Almost all these compounds were previously identified in the composition of the species [6,11,12,13,14,22,25,49,53]. The novelty of the present study consists in the identification and quantification of ellagic, salycilic, and carnosic acids, together with naringenin and vitexin, which, to the best of our knowledge, are reported for the first time in this study. There is important variation in the secondary metabolites of *D. moldavica*, related to its growth period, ecological conditions, and geographical location [54]. The *D. moldavica* species can be cultivated in ecological systems with favorable micro-climates and optimized technological conditions [3]. Exposure to different environmental conditions can increase the production of reactive oxygen species (ROS), and, in this way, cultivation treatments that are used for this species may affect the quality of obtained plants. By producing secondary metabolites with antioxidant activity, the vegetal material may participate in the detoxification of ROS [46,47]. Flavonoids and polyphenols, in general, play an important role in the defense of the species against the damaging effects of ROS and in reducing their generation [39,46,54].

Regarding antioxidant activity, existing studies link it to essential oil composition [1,8,18,19,40,55], but there are studies that link it to phenolic composition [6,11,12,13,19,24,27]. It is widely known that this activity may be one of the most important mechanisms forming the basis of numerous biological activities, such as those that are cytotoxic or antibacterial [12,22]. Most of the similar studies that have been performed used the same methods to assess the antioxidant capacity of the species. The results obtained in these studies reported significant activity of the methanolic [12] or aqueous [13] extracts of the species, but with significantly higher values in the case of DPPH assay and significantly lower values in the case of FRAP assay. When comparing the solvents used for extraction, it appears that an ethanol–methanol (1:1) mixture, followed by methanol [6] and ethyl acetate [11,24] was proved to be the most effective at extracting the compounds responsible for the antioxidant capacity of the species. The most effective extraction method for increasing the antioxidant capacity of the species (assessed using the DPPH method) seems to be extraction using supercritical fluid [51]. The cultivation treatments used for this species show that plants treated with significant concentrations of ammonium in the nutrient solution showed significantly higher levels of antioxidant capacity [49]. In all cases, the values obtained in the present study are significantly higher than the ones reported in similar studies.

Due to the high abundance and availability of polyphenols from *D. moldavica*, they have been the subject of numerous investigations for their cytotoxic and anticancer effects [14].

Because of its complex chemical composition characterized by a high abundance and availability of polyphenols, several studies have aimed to investigate the biological properties of its isolated compounds [14,56]. Their cytotoxic and anticancer potential was met with particular interest. In this regard, recent studies have described the cytotoxic mechanisms of *D. moldavica* constituents such as flavones, flavonols, lignans, caffeic acid derivatives [14], tilianin [56], and geraniol [10]. The chemoprotective effect of flavonoids was demonstrated against multiple myeloma and acute myeloid leukemia [14]. Tilianin, identified as the bulk of the total flavonoids extracted from *D. moldavica*, was suggested as a possible choice for use in developing immunotherapeutic agents based on its in vitro ability to inhibit human pharyngeal squamous cell carcinoma. A comprehensive evaluation indicated not only the anti-tumor potency of tilianin on human pharyngeal squamous carcinoma (FaDu) cells and intrinsic apoptotic signaling pathways, but also its role in dendritic cell maturation as a beneficial response towards the tumor’s immunosuppressive microenvironment [56]. Geraniol, a cyclic monoterpene with recognized antioxidant efficacy, exhibits significant antitumoral effects against various types of cancers [9].

Our results are in agreement with previous studies presenting the in vitro antimicrobial potential of *D. moldavica*-derived products, especially when evaluated against Gram-positive bacteria [22]. The available literature mostly underlines *D. moldavica*’s essential oils as valuable alternatives to classical antibiotics [1,18]. Aćimović et al. reported more intense antioxidant and antimicrobial activities against food-borne pathogens such as *Staphylococcus aureus*, *Escherichia coli*, *Salmonella Typhimurium*, and *Listeria monocytogenes* from essential oils than hydrolate, a distillation process byproduct [1].

Relatively little of the available information refers to ethanolic extracts. An ethanolic extract obtained from *D. moldavica* cultivated in Iran was reported for its in vitro ability to inhibit the antibiotic-resistant *Escherichia coli* and *Klebsiella pneumoniae* nosocomial strains [21]. The ethyl acetate fraction of *D. moldavica* L. ethanolic extract demonstrated efficacy against *S. aureus* isolates with an established minimal inhibitory concentration (MIC) of 50% and 90% for 780 μg/mL and 1065 μg/mL, respectively, and an ability to disrupt biofilm formation from cell membrane damage. The same product was described as having significant antibacterial activity against other Gram-positive bacteria (*S. epidermidis*, *S. haemolyticus*, *E. faecalis*, and *E. faecium*) in a dose-dependent manner, but no inhibitory effects against Gram-negative bacteria or fungi [22].

Regarding the cytotoxicity assays, a similar trend toward a lack of toxicity on fibroblasts was revealed for a methanolic *D. moldavica* extract, but a dermal fibroblast cell line was the subject of the study [12]. Other reports have revealed a cytotoxic effect of this species on myeloma cell lines (KMS-12-PE) and AML (Molm-13) in relation with the polyphenols in its composition [14]. Similarly, ethyl acetate extracts of the species exhibited significant cytotoxic effects on human epidermal keratinocytes (HaCaT) [22], on two human cancerous cell lines, SW- 48 and MCF- 7 [11], and on HeLa, A549, and HepG2 cells [32]. It is, therefore, clear that the present study brings novelty and originality by using novel cell lines, BJ (fibroblast), and DLD-1 (colorectal adenocarcinoma).

Among the three investigated *D. moldavica* L. cultivars, B2 was characterized by the highest amounts of TFC, TPC, and TPAC and presented the most intense biological potential. To better explore the relationship between the chemical groups and the potency of their biological properties, Pearson coefficients were calculated. Their values pointed out a significant positive correlation between FRAP assay and both TPAC (*r^2^* = 0.987, *p* < 0.05) and TPC (*r^2^* = 0.951, *p* < 0.05), respectively. Thus, for the tested extracts, the antioxidant activity evaluated using FRAP assay was found to be connected to the total phenolic acid and polyphenol content.

Based on the phytochemical profile evaluation, the B2 sample proved to be the richest source of TFC, TPC, TPAC and of the polyphenolic individual components. Furthermore, this sample proved to be the most effective in terms of its biological potential, exhibiting the most significant antioxidant, antimicrobial, and cytotoxic activities. Its level of selective cytotoxicity against the colorectal adenocarcinoma cell line and its lack of toxicity against normal cells are aspects that need to be highlighted. These findings bring additional arguments for strongly considering the *D. moldavica* L. B2 cultivar as a promising vegetal product, which warrants further investigation aimed to develop vegetal medicinal drugs.

## 4. Materials and Methods

### 4.1. Chemicals and Reagents

A CCK-8 assay reagent, a Minimum Essential Medium Eagle (MEM), and an RPMI-1640 medium were purchased from Sigma-Aldrich (St. Louis, MO, USA). The reagents that were added to cell cultures, such as the 1% antibiotic/antimycotic and 10% fetal bovine serums, were purchased from Gibco Life Technologies (Paisley, UK) and from EuroClone (MI, Pero, Italy). Bacterial reference strains were obtained from Oxoid Ltd. (Hampshire, UK), and culture mediums, such as Mueller–Hinton broth and Mueller–Hinton agar, were purchased from Merck (Darmstadt, Germany). All other solvents and reagents were of analytical-grade purity and were purchased from Merck KgaA (Darmstadt, Germany). The compounds used as references in the LC–MS method were also of analytical-grade purity and were purchased from Phytolab (Vestenbergsgreuth, Germany).

### 4.2. Cultivation Conditions

The three cultivars were obtained from a culture initiated in 2020 in the experimental fields of the Agro-Botanical Garden of the University of Agricultural Sciences and Veterinary Medicine (UASVM) in Cluj-Napoca, Romania (latitude 46°45′36″ N and longitude 23°34′24″ E, elevation 380–430 m). The type of oil in the experimental fields in Cluj-Napoca is luvisol. An analysis of soil samples (depth 0–20 cm) from the experimental plot was conducted at an authorized agrochemical laboratory, the Office of Pedological and Agrochemical Studies Cluj-Napoca. According to the performed analysis, the soil has a clay loam texture (11.45% coarse sand, 32.96% fine sand, 9.15% silt I, 13.20% silt II, and 33.24% clay), a slight alkaline pH, a moderate carbonate content, a moderate humus level, a low nitrogen content, and very good/high phosphorus and potassium levels compared to reference thresholds from the literature (Table 6) [57].

**Figure 4:**
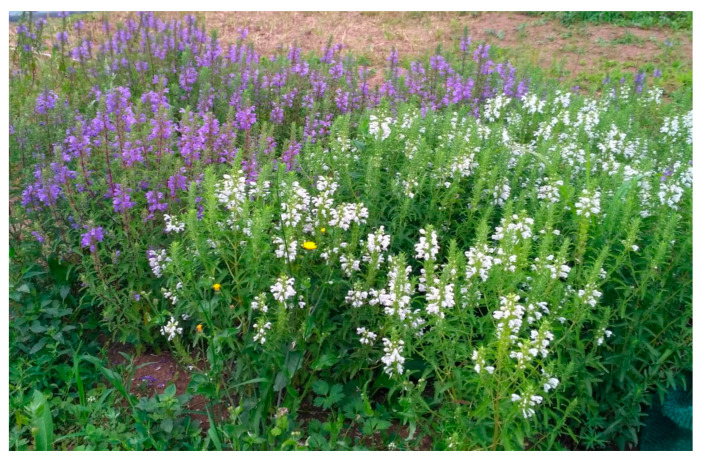
The aspect of *D. moldavica* cultivars before harvesting.

The three cultivars and their proveniences are as follows:

- A1—Aroma 1, created in 1991 at the Institute of Genetics, Physiology, and Plant Protection, Chisinau (Republic of Moldova) (https://igfpp.md/certificate-soi-planta, accessed 17 November 2019), with blue flowers;
- A2—Aroma 2, created at the previously mentioned institution, with white flowers;
- B2—Buzău 2, created at the Vegetable Research–Development Station, Buzău‚ with blue flowers (Figure 4).

The culture was obtained using the block method, with each variant in 3 repetitions and each experimental plot having an area of approximately 3 m^2^. The seedlings produced in the greenhouse were planted. There was a planting distance of 50 cm between rows and 20 cm between plants per row (10 plants/m^2^). The experimental plots were manually maintained by weeding. On 24 July 2020, the blooming aerial parts of the plants were harvested with a sickle. The average height of the plants was 75 cm for A1, 71 cm for A2, and 73 cm for B2. The harvested plant material was weighed and then dried in a shady and dry place. After drying for 18 days, the dry material was weighed again and the drying yield was calculated (Table 7) [42].

### 4.3. Preparation of Extracts

The vegetal material in the aerial part of the plants was collected during the full blooming period. The vegetal material was identified by Lecturer Irina Ielciu, Ph.D., and voucher specimens were deposited at the herbarium of the Pharmacognosy Department of the Faculty of Pharmacy Cluj-Napoca (voucher no. 563). The aerial parts were air-dried after collection and grounded using a Grindomix GM 200 knife mill (Éragny, France). Subsequently, the obtained powder was macerated for 10 days with 70% *v*/*v* ethanol in water and 2–3 shakes/day, and the obtained ethanolic extract was filtered at the end of the maceration period [58,59,60,61].

### 4.4. Total Polyphenolic, Flavonoid, and Phenolic Acid Content

The total phenolic content was spectrophotometrically evaluated using a Folin–Ciocâlteu reagent according to the recommendations of *the European Pharmacopoeia* [62]. The values of TPC were calculated using a calibration curve of gallic (R^2^ = 0.9942) and rosmarinic acids (R^2^ = 0.9999) and expressed as mg of gallic acid equivalent (GAE)/100 g of dry vegetal material and mg of rosmarinic acid equivalent (RAE)/100 g of dry vegetal material. The quantitative determination of total flavonoid content was obtained spectrophotometrically using a method with aluminum chloride. TFC values were calculated using a calibration curve of rutoside (R^2^ = 0.9952) and expressed as mg of rutoside equivalent (RE)/100 g of dry vegetal material. The level of phenolic acid content was determined spectrophotometrically using a method described in the 10th Edition of *the Romanian Pharmacopoeia* (*Cynarae folium* monograph). TPAC results were expressed as mg of rosmarinic acid equivalent (RAE)/g of dry vegetal material, after being calculated using a rosmarinic acid calibration curve graph (R^2^ = 0.9991). All experiments were performed in triplicate [58,59,63,64,65]. The values of all absorbances were measured using a UV–VIS spectrophotometer (Specord 200 Plus, Analytik Jena, Jena, Germany).

### 4.5. LC–MS Analysis

#### LC–MS Apparatus

The LC–MS analysis was performed using a Shimadzu Nexera I LC–MS-8045 (Kyoto, Japan) UHPLC system, equipped with a quaternary pump, an autosampler, an ESI probe, and a quadrupole rod mass spectrometer. Separation was carried out on a Luna C18 reversed phase column (150 mm × 4.6 mm × 3 µm, 100 Å, Phenomenex-Torrance, CA, USA). The column’s temperature was set at 40 °C during the analysis. The mobile phase consisted of a gradient of LC–MS analytical-grade methanol and ultrapurified water prepared using the Simplicity ultrapure water purification system (Merck Millipore, Billerica, MA, USA) and having the composition described in Table 8. LC–MS analytical-grade formic acid was used as the organic modifier of the mobile phase. The flow rate was maintained at 0.5 mL/min during the analysis (total analysis time: 35 min).

Detection was performed using a quadrupole rod mass spectrometer with electrospray ionization (ESI), both in negative and positive multiple reaction monitoring (MRM) ion modes. The temperature was set at 300 °C. Nitrogen with 30 psi and a flow rate of 10 L/min was used for vaporization and as the drying gas. The capillary potential was set at +3000 V.

Injection volumes were set at 1 μL for references and 10 μL for each sample. Identification was carried out via a comparison of the retention times, MS spectra, and the transitions between the separated compounds and references. Identification and quantification were based on the main transition from the MS spectra of each individual compound. Calibration curves (R^2^ = 0.9964–0.9999) were plotted for the quantification of compounds and references. This method was validated by assessing its linearity, precision, and accuracy according to the International Conference on Harmonization guidelines (ICH). The LOD and LOQ were calculated after the injection of a series of different concentrations into each analyzed compound. The accuracy of this method was determined in duplicate using a recovery experiment. The tested samples were injected in triplicate [66,67].

### 4.6. Antioxidant Capacity Assays

#### 4.6.1. DPPH Radical Scavenging Activity

DPPH bleaching assay is a spectrophotometric method based on the reaction of DPPH reagents and antioxidants, and it was performed by adding 2 mL of each sample at different concentrations to 2 mL of a DPPH methanolic solution at a concentration of 0.1 g/L. The mixture was maintained at 40 °C in a thermostatic bath for 30 min. Absorbance (A) was measured at 517 nm. The inhibition of the DPPH (I) radical was calculated using the following formula: I% = (A control − A sample/A control) × 100, where A control is the absorbance of the control, composed of the DPPH radical solution–methanol mixture (which contains all reagents except the sample), and A sample is the absorbance of the DPPH radical solution–sample mixture. The DPPH radical scavenging activity of the sample was expressed as IC_50_ (µg/mL), the concentration required to cause 50% DPPH inhibition (Figures S18–S21). The assays were performed in triplicate [64,65,68,69].

#### 4.6.2. Ferric-Reducing Antioxidant Power Assay (FRAP)

The FRAP method is based on the changes to the color of a complex of a 2,4,6-tri(2-pyridyl)-1,3,5-triazine (TPTZ) radical and an Fe^3+^ ion, assessed using the reduction of the ferric ion to the ferrous ion (Fe^2+^) in the complex [70]. The FRAP reagent used was a mixture of 2.5 mL of 10 mM TPTZ solution in 40 mM HCl, 2.5 mL of 20 mM ferric chloride solution, and 25 mL of acetate buffer with a pH of 3.6. A total of 4 mL from each sample was diluted to 1.8 mL with water and mixed with 6 mL of the FRAP reagent. A blank solution was prepared using water instead of the sample. The antioxidant capacity of each sample was assessed in correlation with the color changes by measuring absorbance at 450 nm. Trolox was used as a reference with a calibration curve (R^2^ = 0.9983). Results were expressed as µM of Trolox equivalent/g of dry plant material. All assays were performed in triplicate [60,71].

### 4.7. Antimicrobial Assays

To screen the in vitro antimicrobial properties of the tested samples, the agar well diffusion assay, a modified EUCAST (European Committee on Antimicrobial Susceptibility Testing) disk-diffusion method, was employed [72]. Four bacterial reference strains obtained from Oxoid Ltd. (Hampshire, UK) were included, namely *Staphylococcus aureus* ATCC 25,923 (methicillin-susceptible *S. aureus*, MSSA), *Staphylococcus aureus* ATCC 700,699 (methicillin-resistant *S. aureus*, MRSA), *Escherichia coli* ATCC 25,922, and *Pseudomonas aeruginosa* ATCC 27853. The bacteria were cultured on Mueller–Hinton (MH) broth and agar, mediums that were purchased from Merck (Darmstadt, Germany, catalogue number 70,192 and 70191-500G). Bacterial inoculums were prepared for each strain by suspending 24 h of pure culture in a sterile saline to obtain a 10^6^ colony-forming unit (CFU)/mL, according to the McFarland scale. Following flood inoculation on MH agar plates, wells with six-millimeter diameters (three for each sample) were aseptically cultured into the MH agar and filled with 60 μL of tested extracts. Both negative (70% ethanol in water V/V) and positive controls (standard antibiotics disks—gentamicin (10 µg), amoxicillin–clavulanic acid (20–10 µg) from Oxoid Ltd., Hampshire, UK, catalogue number CT0794B and CT0223B) were considered. After 24 h of incubation at 37 °C, the growth inhibition zone diameters (in mm) were measured (Figure S22). The same inoculums were used when performing the broth microdilution method, which allowed us to establish the minimum inhibitory (MIC) and bactericidal (MBC) concentrations. Additionally, 96-well bottom “U” polystyrene plates were used to prepare two-fold serial dilutions of the tested extracts in 100 µL of MH broth. The resulting concentrations of A1, A2, and B2 (ranging from 0.085 to 2.750 μmol of GAE/100 μL, 0.089 to 2.850 μmol of GAE/100 μL and 0.103 to 3.300 μmol of GAE/100 μL, respectively) were cultured with a 5.0 µL bacterial inoculum for 24 h at 37 °C. The MICs values were considered the lowest concentrations able to inhibit visible bacterial growth (i.e., no turbidity in the well) compared to the negative control (MH broth). A reading of MBCs values was performed following the 24 h culture on MH agar and produced a result of 10.0 µL from each well; this was done by recording the lowest concentrations associated with no visible bacterial growth on the agar plates. MH broth was also tested as an MIC negative control. Furthermore, the MIC index, based on the ratio of MBC/MIC, indicated whether the extract displayed bactericidal (MBC/MIC ≤ 4) or bacteriostatic (MBC/MIC > 4) effects against the tested bacterial strains [61,66,73,74].

### 4.8. Cytotoxicity Assay

The cytotoxic potential of the samples was tested on a normal diploid foreskin fibroblasts cell line (BJ cells, ATCC CRL-2522) and a colorectal adenocarcinoma cell line (DLD-1, ATCC CCL-221), obtained from the “Prof. Dr. Ion Chiricuţă” Institute of Oncology (Cluj-Napoca, Romania). The BJ cells were maintained in an MEM medium, supplemented with a 10% fetal bovine serum and a 1% antibiotic/antimycotic, while the DLD-1 cells were cultured in an RPMI-1640 medium, supplemented with a 10% fetal bovine serum and a 1% antibiotic/antimycotic. The cells were incubated at 37 °C in a humidified atmosphere, supplemented with 5% of CO_2_. Suspensions of cells with a concentration of 5 × 10^3^ cells/well were seeded in 96-well tissue culture plates in their propagation medium. After 24 h of incubation, the cultures were exposed to different concentrations of each sample, calculated based on the determined TPC and expressed as µmol of GAE as follows: 0.142–0.714 µmol of GAE for the A1 sample, 0.137–0.687 µmol of GAE for the A2 sample, and 0.165–0.827 µmol of GAE for the B2 sample. Controls for viability were the 100% untreated cells (negative control), the solvent of extraction (70% ethanol in water *v*/*v*), and cytotoxicity control (positive control—cisplatin 25 µM). Each cell’s viability was measured using a cell counting kit-8 (CCK-8) assay (Sigma–Aldrich, St. Louis, MO, USA) following the manufacturer’s protocol. Subsequently, after 24 h, the CCK-8 solution was added to each well and the plates were incubated for an additional 1.5 h. Optical density was measured at 450 nm using a BioTek Synergy 2 microplate reader (Winooski, VT, USA). The results are expressed as percent of viability relative to the negative control (untreated cells). The IC_50_ (concentration that inhibits cell growth by 50%) values of the selected extracts (A1, A2, and B2) against the DLD-1 cell line were expressed using a nonlinear regression of concentration–response curves. All experiments were performed in triplicate [58,66,73,75].

### 4.9. Statistical Analysis

Results of this study are presented as mean ± standard deviation (SD) for each type of the performed assays, with the data statistically analyzed using the ANOVA GraphPad Prism software, version 6.0 (San Diego, CA, USA). A one-way analysis of variance (ANOVA) was conducted, followed by Tukey’s post hoc test, to determine the statistical significance between the means of the three main phytochemical groups of ethanolic extracts, namely total polyphenolic (TPC), flavonoid (TFC), and phenolic acid (TPAC) content and their antioxidant, antimicrobial, and cytotoxicity property assays. A *p*-value below 0.05 was considered statistically significant. Additionally, to evaluate the relationship between biological activity and chemical composition, Pearson’s correlation coefficients were calculated using the CORREL function [66,71,73].

## 5. Conclusions

The superior quality of the vegetal raw material obtained through culture is reflected by the higher content of polyphenolic compounds associated with important antioxidant and antimicrobial properties. Furthermore, our results highlighted significant cytotoxic potential that was exhibited on the colorectal adenocarcinoma cell line. Antimicrobial potential was proved, especially towards Gram-positive bacteria, and its connection with antioxidant capacity was established for both the cytotoxicity assays and the antimicrobial ones with the polyphenolic compositions of the three tested cultivars. Among these cultivars, the one that proved to have the richest polyphenolic composition and the most significant biological potential was the B2 sample. These findings offer additional arguments for strongly considering *D. moldavica* L. cultivars (especially the ones bearing blue flowers) as promising vegetal products, warranting further investigation aimed to elucidate the mechanisms of these biological activities in order to prove the biological potential of this species as a vegetal medicinal product.

## Figures and Tables

**Table 1:** Total polyphenolic, flavonoid, and phenolic acid content of *D. moldavica* samples.

Sample	TPC (g RAE/100 g Dry Plant Material)	TPC (g GAE/100 g Dry Plant Material)	TFC (g RE/100 g Dry Plant Material)	TPAC (g RAE/100 g Dry Plant Material)
A1	4.644 ± 0.135	4.862 ± 0.163	1.218 ± 0.096	2.407 ± 0.261 *
A2	4.474 ± 0.115	4.620 ± 0.151	1.100 ± 0.063	2.116 ± 0.176
B2	5.363 ± 0.157 **	5.631 ± 0.175 **	1.255 ± 0.167	2.711 ± 0.414 **

Note: Each value represents the mean ± standard deviations of three independent measurements. RAE—rosmarinic acid equivalents; GAE—gallic acid equivalents; RE—rutin equivalents. * *p* < 0.05 for B2 vs. A2 and A1 vs. A2; ** *p* < 0.001 for B2 vs. A1 and A2.

**Table 2:** The identified and quantified components of *D. moldavica* samples (mg/mL extract) collected using LC–MS analysis.

Name of Reference or Separated Compound	Reference		Separated Compound		Samples		
	Retention Time (min)	Main MS Transition	Retention Time (min)	Main MS Transition	A1 Content (mg/mL)	A2 Content (mg/mL)	B2 Content (mg/mL)
Caffeic acid	13.5	179.0 > 135.0	13.7	179.0 > 135.0	0.500 ± 0.0163	0.477 ± 0.0262	0.527 ± 0.0330
Chlorogenic acid	11.9	353.0 > 191.0	12.0	353.0 > 191.0	0.287 ± 0.0287	0.237 ± 0.0249	0.273 ± 0.0125
*trans-p*-coumaric acid	17.4	163.0 > 119.0	17.5	163.0 > 119.0	0.130 ± 0.0216	0.107 ± 0.0125	0.177 ± 0.0287
Ellagic acid	27.2	301.0 > 185.0	26.9	301.0 > 185.0	0.597 ± 0.0411	0.393 ± 0.0368	0.147 ± 0.0205
Ferulic acid	18.4	193.0 > 134.0	18.5	193.0 > 134.0	0.012 ± 0.0021	0.014 ± 0.0022	0.013 ± 0.0017
Rosmarinic acid	21.4	358.9 > 161.0	21.3	358.9 > 161.0	5.833 ± 0.0624	5.337 ± 0.0411	6.320 ± 0.0535
Salicylic acid	23.5	137.0 > 93.0	23.4	137.0 > 93.0	0.098 ± 0.0155	0.098 ± 0.0155	0.085 ± 0.0041
Carnosic acid	32.0	331.2 > 285.1	30.6	331.2 > 285.1	0.098 ± 0.0165	0.098 ± 0.0165	0.098 ± 0.0165
Carnosol	30.6	329.1 > 285.1	30.4	329.1 > 285.1	0.003 ± 0.0005	0.003 ± 0.0005	0.003 ± 0.0005
Apigenin	28.1	269.0 > 117.0	28.1	269.0 > 117.0	0.967 ± 0.0492	0.487 ± 0.0492	0.767 ± 0.0386
Hyperoside	20.3	463.1 > 300.0	20.6	463.1 > 300.0	0.068 ± 0.0040	0.370 ± 0.0327	0.038 ± 0.0033
Luteolin	26.8	287.0 > 153.0	26.8	287.0 > 153.0	0.019 ± 0.0017	0.010 ± 0.0012	0.020 ± 0.0041
Luteolin-*7-O*-glucoside	19.9	447.0 > 284.9	19.8	447.0 > 284.9	0.820 ± 0.0327	0.787 ± 0.0330	0.667 ± 0.0655
Naringenin	26.2	271.0 > 119.0	26.2	271.0 > 119.0	0.004 ± 0.0005	0.004 ± 0.0005	0.005 ± 0.0005
Quercetin	25.4	300.9 > 151.0	25.4	300.9 > 151.0	0.003 ± 0.0009	0.006 ± 0.0008	0.003 ± 0.0008
Rutoside	20.2	609.0 > 300.0	20.3	609.0 > 300.0	0.068 ± 0.0029	0.062 ± 0.0053	0.049 ± 0.0054
Vitexin	18.4	431.0 > 311.0	18.5	431.0 > 311.0	0.004 ± 0.0005	0.004 ± 0.0005	0.003 ± 0.0005

Note: Values represent the mean ± standard deviations of three independent measurements.

**Table 3:** Antioxidant activity of *D. moldavica* samples.

Sample	DPPH IC_50_ (µg/mL)	FRAP µmol Trolox Equivalent/g Dry Plant Material
A1	40.901 ± 0.161	293.194 ± 0.213
A2	35.542 ± 0.043 **	301.493 ± 0.115
B2	35.650 ± 0.063 **	330.165 ± 0.754 **
Ascorbic acid	5.691 ± 0.123	-

Note: Values represent the mean ± standard deviations of three independent measurements; ** *p* < 0.001 for DPPH—A2 and B2 vs. A1, and FRAP—B2 vs. A1 and A2.

**Table 4:** In vitro antibacterial activity of the *D. moldavica* samples collected using the well diffusion method.

Zone of Inhibition (mm)				
Sample	MSSA	MRSA	*Escherichia coli*	*Pseudomonas* *aeruginosa*
A1	19.67 ± 0.52	17.33 ± 0.52	15.50 ± 0.55	0
A2	20.17 ± 0.41	18.17 ± 0.41	15.83 ± 0.41	0
B2	23.50 ± 0.55 ^a, b, d^	20.50 ± 0.55 ^a, b, d^	17.33 ± 0.52	0
Amoxicillin–clavulanic acid	29 ± 0.00 ^a, b, c^	28 ± 0.00 ^a, b, c^	19 ± 0.00 ^a, b, c^	0
Gentamicin	20 ± 0.00	17 ± 0.00	19 ± 0.00	18 ± 0.00

Note: MSSA—methicillin-susceptible *Staphylococcus aureus*; MRSA—methicillin-resistant *Staphylococcus aureus*; values represent the mean ± standard deviations of two independent measurements. ^a–d^ Means with different subscript letters within a row are significantly different at *p* < 0.05. A1 (4.862 mg GAE/mL); A2 (4.620 mg GAE/mL); B2 (5.631 mg GAE/mL). Antibiotic disks: amoxicillin–clavulanic (20–10 µg); gentamicin (10 µg).

**Table 5:** In vitro antibacterial activity of the *D. moldavica* samples collected using broth microdilution assay.

MIC Index MBC (μmol GAE/100 μL)/MIC (μmol GAE/100 μL)			
Sample	MSSA	MRSA	*Escherichia coli*
A1	1 0.356/0.356	2 0.712/0.356	1 0.712/0.712
A2	2 0.343/0.171	4 0.687/0.171	4 2.750/0.687
B2	2 0.825/0.412	4 0.825/0.206	4 3.300/0.825

**Table 6:** Soil characteristics of the experimental plot.

Parameters of Analysis	Analytical Methods	Values
pH	Potentiometric	8.05
Humus	Walkley–Black	3.24%
Nitrogen	Kjeldahl	0.129%
Phosphorus	Colorimetric	224 ppm
Potassium	Flame photometry	304 ppm
Basic cation saturation	Kappen	20.96 m/100 g
CaCO_3_	Scheibler	10.4 m/100 g
Base saturation (V)	Calculation	92
Granulometric	Kacinscki	clay loam

**Table 7:** The drying yield for the *D. moldavica* cultivars.

Cultivars (Samples)	Average Mass of a Plant (g)		Drying Yield
	Fresh 27 July 2020	Dry 8 August 2020	
A1	223.3	54.1	1/4.13
A2	205.8	51.8	1/3.97
B2	212.5	55.2	1/3.85

**Table 8:** Composition of the LC–MS mobile phase gradient.

Time (min)	% Methanol	% Water	% of 2% Formic Acid in Water
0.00	5	90	5
3.00	15	70	15
6.00	15	70	15
9.00	21	58	21
13.00	21	58	21
18.00	30	41	29
22.00	30	41	29
26.00	50	0	50
29.00	50	0	50
29.01	5	90	5
35.00	5	90	5

## Data Availability

Not applicable.

## Supplementary Materials

The following supporting information can be downloaded at: https://www.mdpi.com/article/10.3390/molecules28041604/s1. Figures S1–S17, LC–MS chromatograms of compounds analyzed from the A2 sample (3 different injections); S18–S21, determination of IC_50_ for the A1, A2, and B2 samples and ascorbic acid; S22, antibacterial activity of the B2 sample using the well diffusion method, S23, antibacterial activity of the A1, A2, B2 samples by well diffusion method against *Pseudomonas aeruginosa*.
