# Supplementary material for: Evaluation of the Cytotoxic, Antioxidative and Antimicrobial Effects of Dracocephalum moldavica L. Cultivars
Source: Molecules. 2023 Feb 7;28(4):1604. doi: 10.3390/molecules28041604 (PMC9965778; doi:10.3390/molecules28041604)
Supplement: Supplementary file 1 [file molecules-28-01604-s001.zip › molecules-2165543-supplementary.pdf]

Supplementary Materials

Evaluation of the Cytotoxic, Antioxidative, and Antimicrobial Effects of *Dracocephalum moldavica* L. Cultivars

Ștefania Simea [1], Irina Ielciu [2,*], Daniela Hanganu [3,*], Mihaela Niculae [4], Emoke Pall [4], Ramona Flavia Burtescu [5], Neli-Kinga Olah [5,6], Mihai Cenariu [4], Ilioara Oniga [3], Daniela Benedec [3], and Marcel Duda [1]

[1] Department of Crop Science, Faculty of Agriculture, University of Agricultural Sciences and Veterinary Medicine Cluj-Napoca, 400372 Cluj-Napoca, Romania
[2] Department of Pharmaceutical Botany, Faculty of Pharmacy, "Iuliu Hațieganu" University of Medicine and Pharmacy Cluj-Napoca, 400010 Cluj-Napoca, Romania
[3] Department of Pharmacognosy, Faculty of Pharmacy, University of Medicine and Pharmacy "Iuliu Hațieganu", 400000 Cluj-Napoca, Romania
[4] Department of Clinical Sciences, University of Agricultural Sciences and Veterinary Medicine Cluj-Napoca, 400374 Cluj-Napoca, Romania
[5] SC PlantExtrakt SRL, 407059 Rădaia, Cluj, Romania
[6] Department of Therapeutical Chemistry, Pharmaceutical Industry and Biotechnologies, Faculty of Pharmacy, Vasile Goldiș Western University from Arad, 310048 Arad, Romania
* Correspondence: dhanganu@umfcluj.ro (D.H.); irina.ielciu@umfcluj.ro (I.I.)

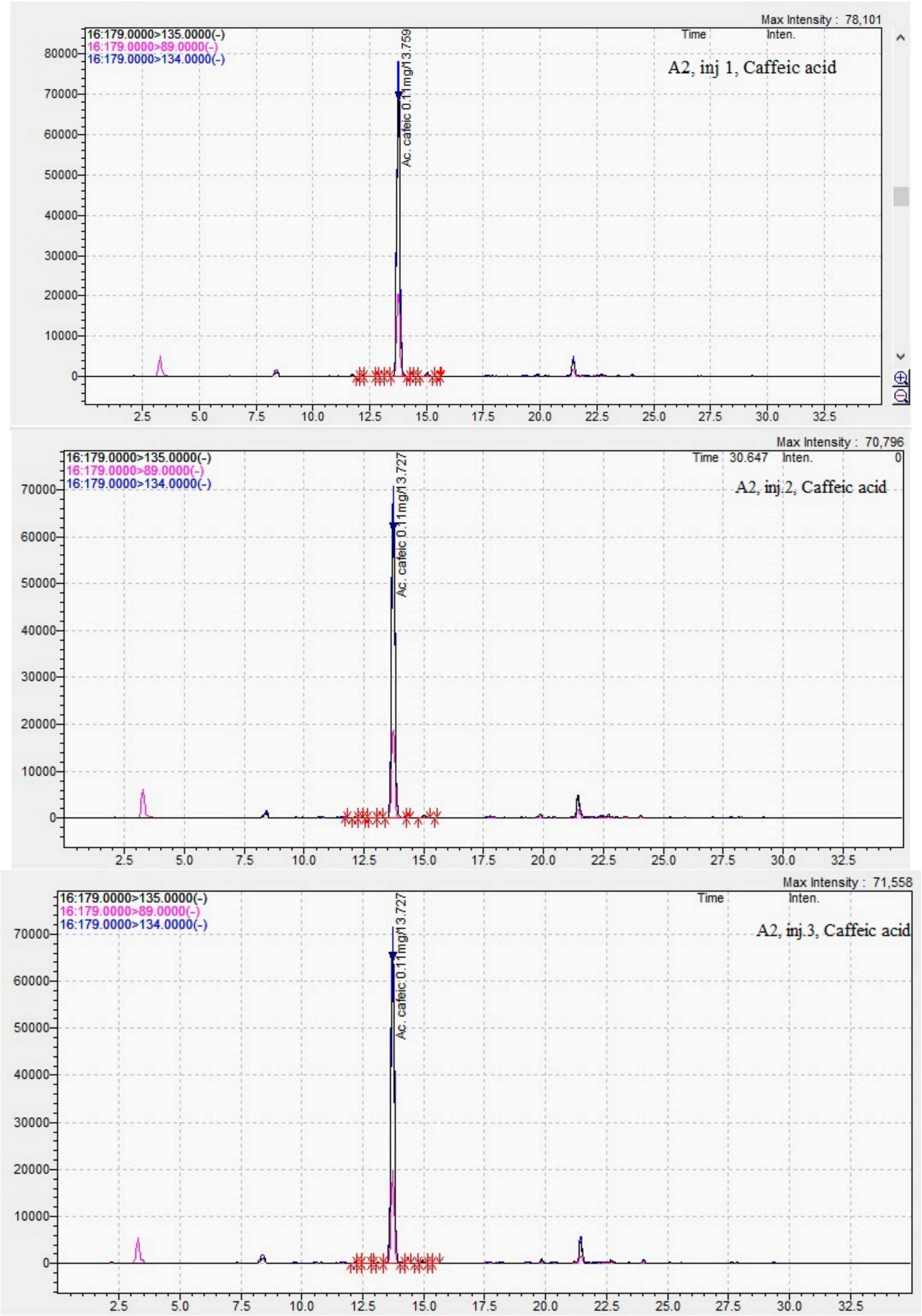

Figure S1. LC-MS chromatograms obtained for the identification of caffeic acid in the A2 sample (3 injections).

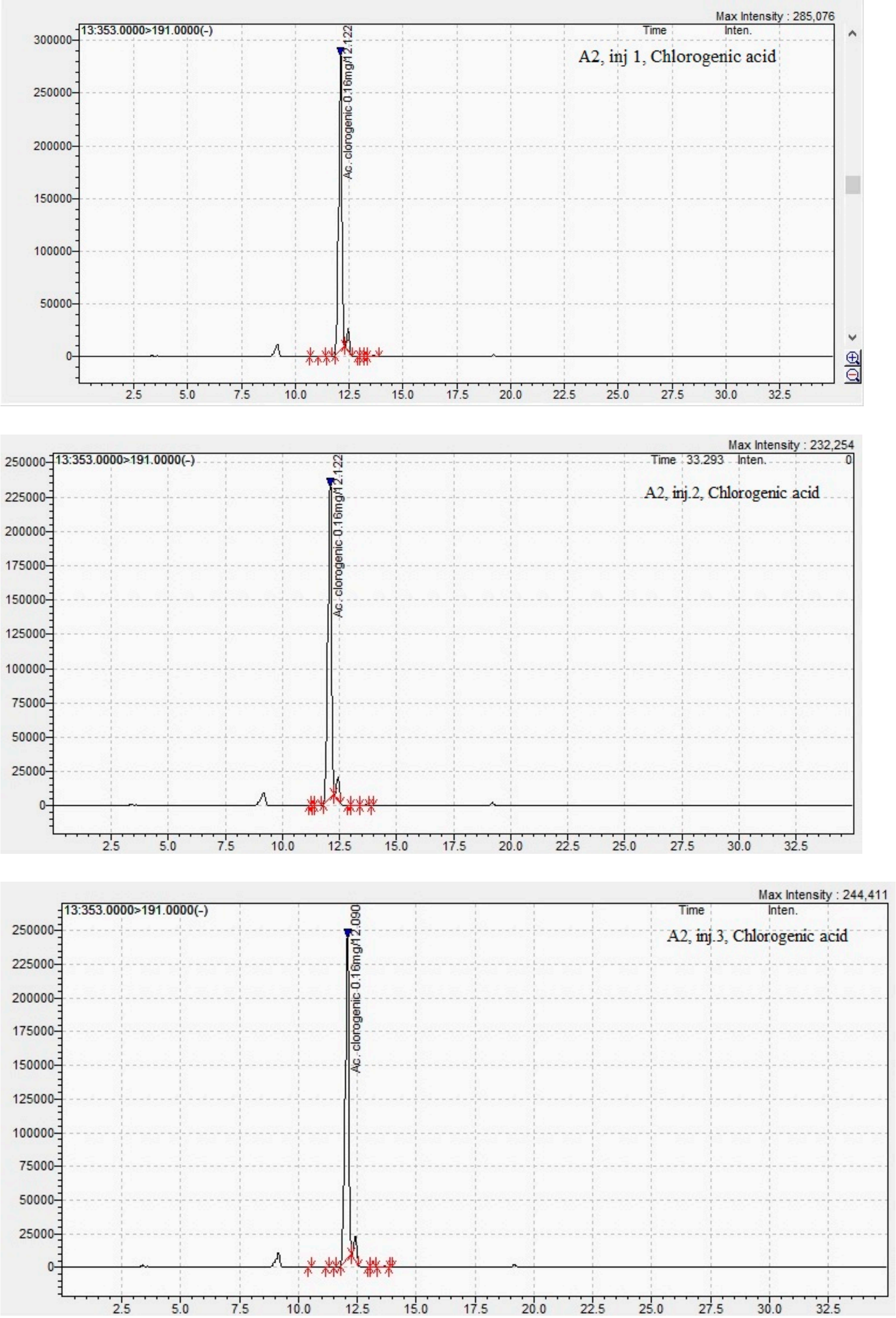

Figure S2. LC-MS chromatograms obtained for the identification of chlorogenic acid in the A2 sample (3 injections).

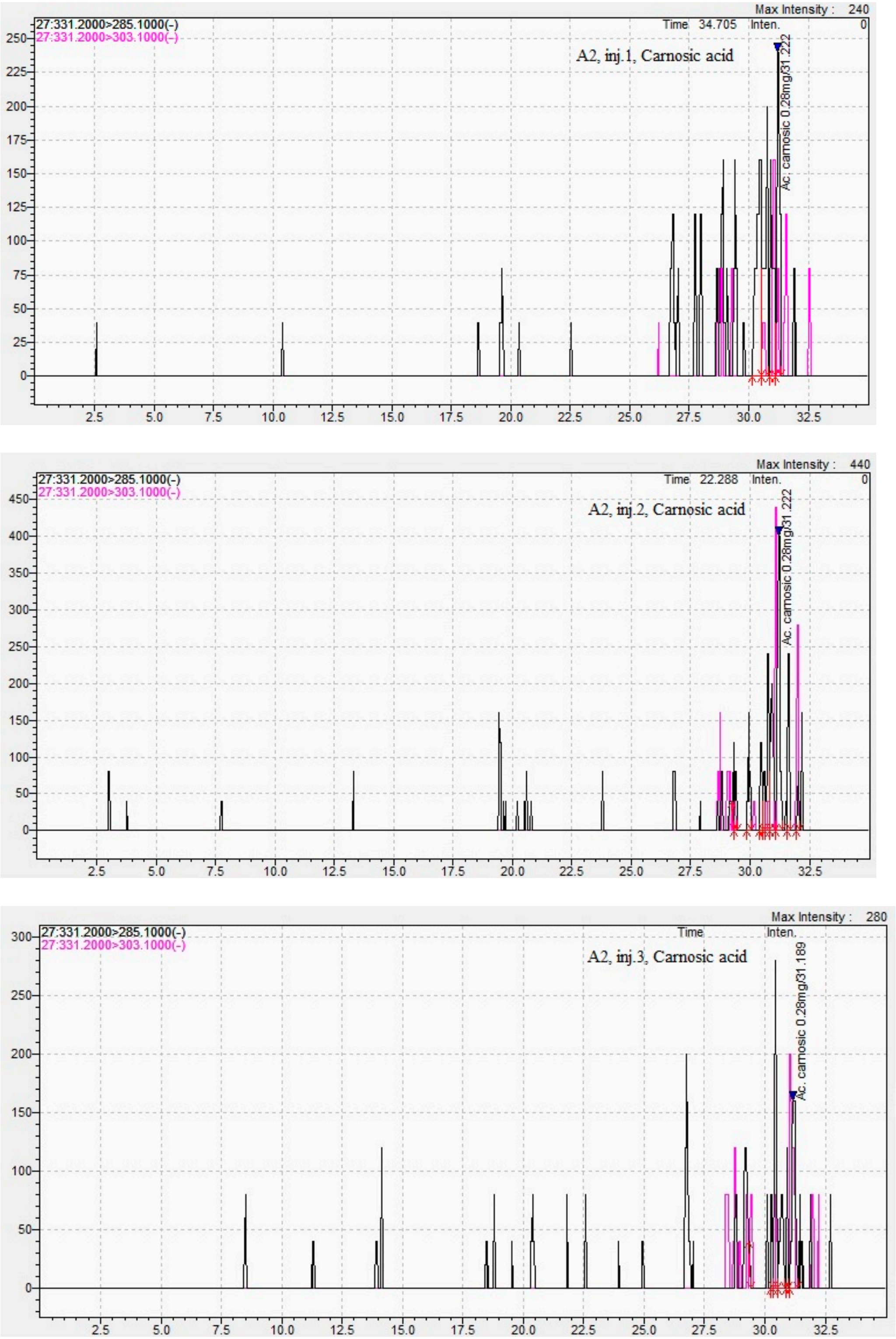

Figure S3. LC-MS chromatograms obtained for the identification of carnosic acid in the A2 sample (3 injections).

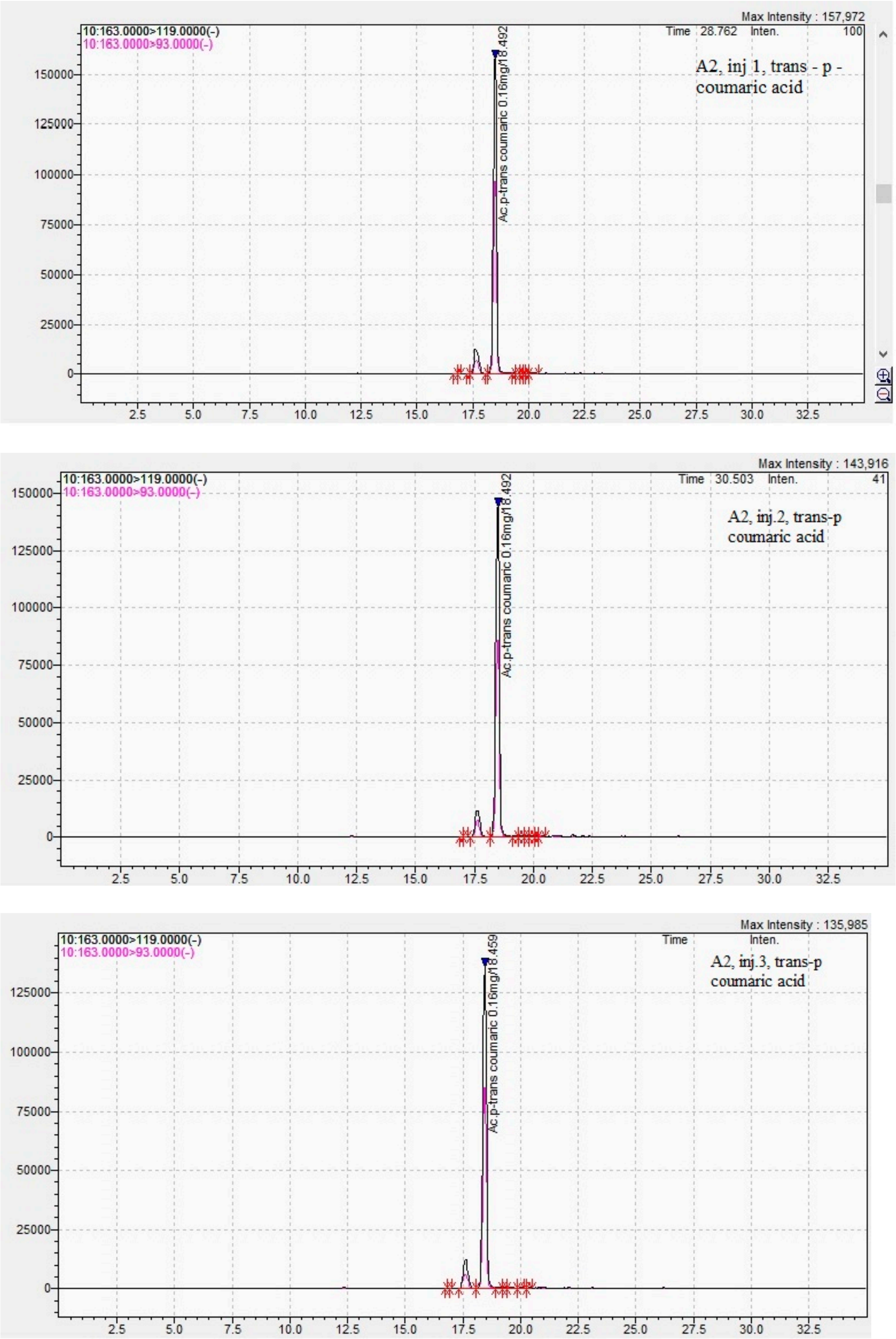

Figure S4. LC-MS chromatograms obtained for the identification of trans-*p*-coumaric acid in the A2 sample (3 injections).

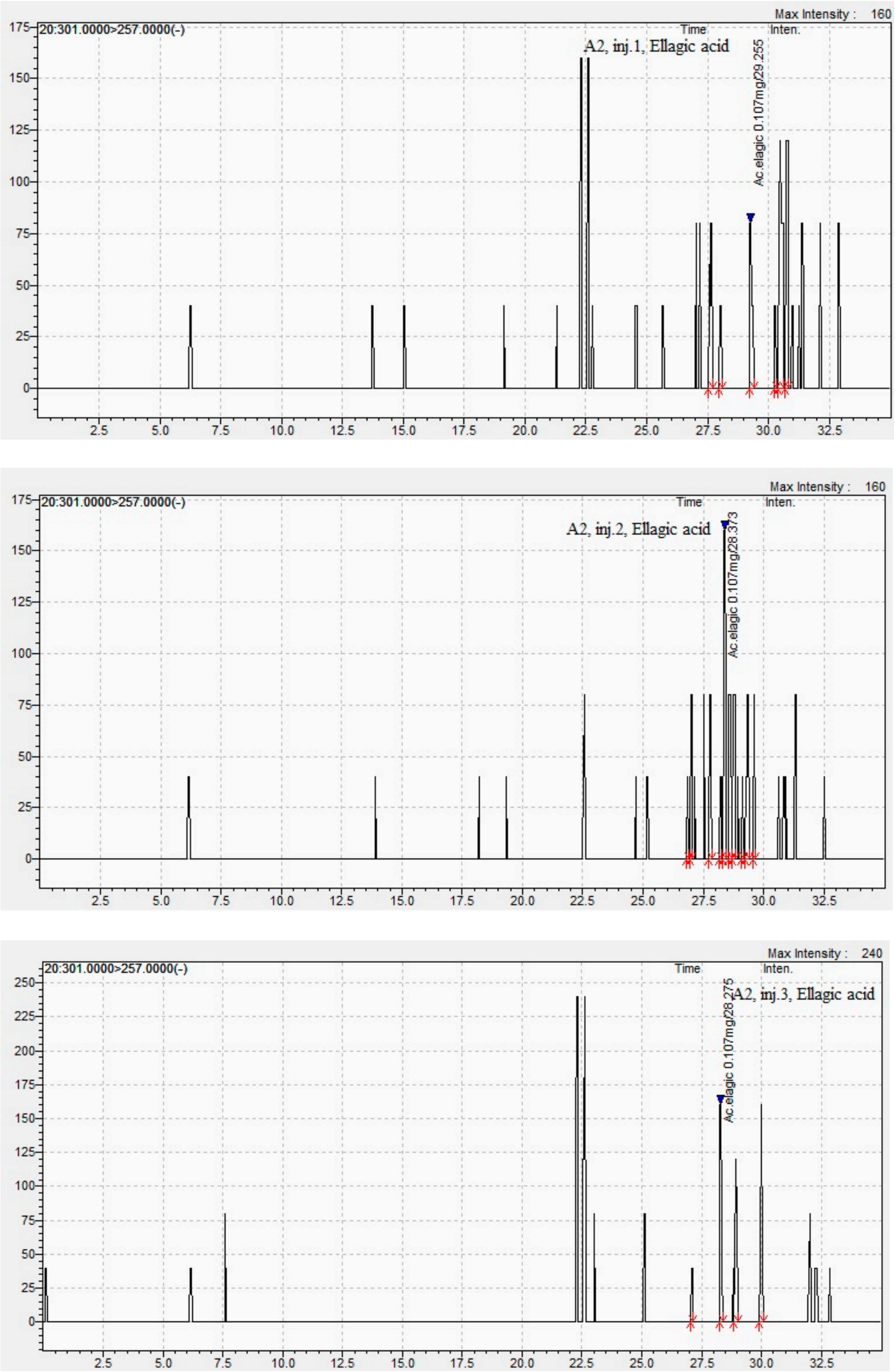

Figure S5. LC-MS chromatograms obtained for the identification of ellagic acid in the A2 sample (3 injections).

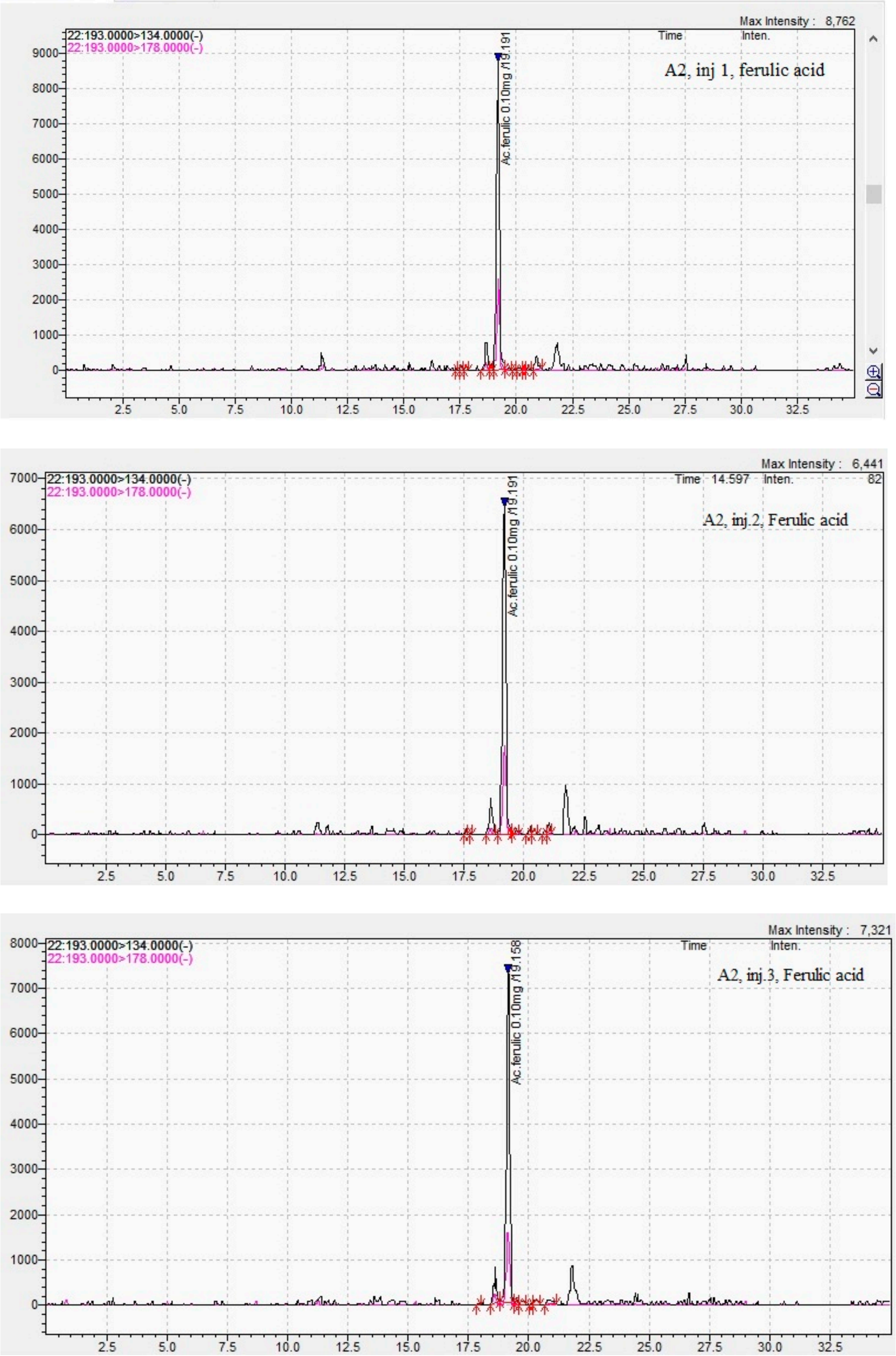

Figure S6. LC-MS chromatograms obtained for the identification of ferulic acid in the A2 sample (3 injections).

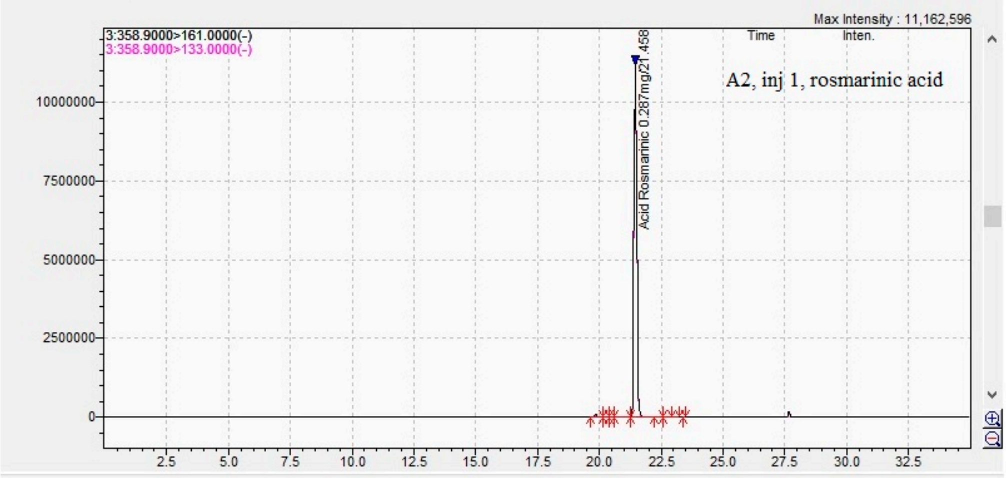

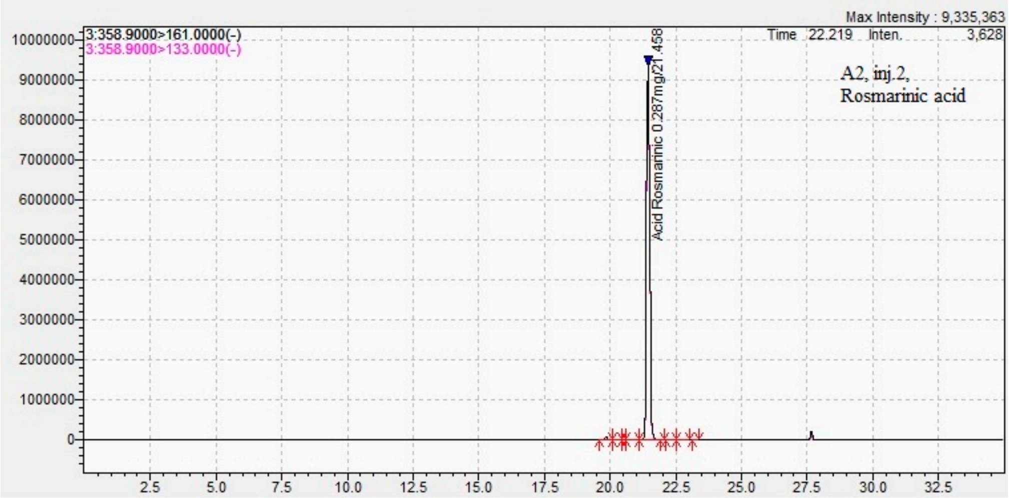

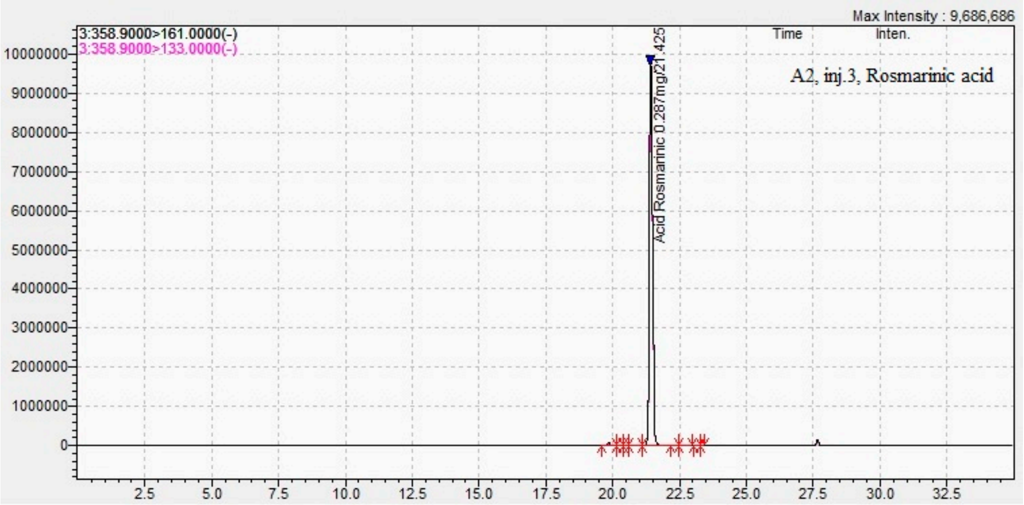

Figure S7. LC-MS chromatograms obtained for the identification of rosmarinic acid in the A2 sample (3 injections).

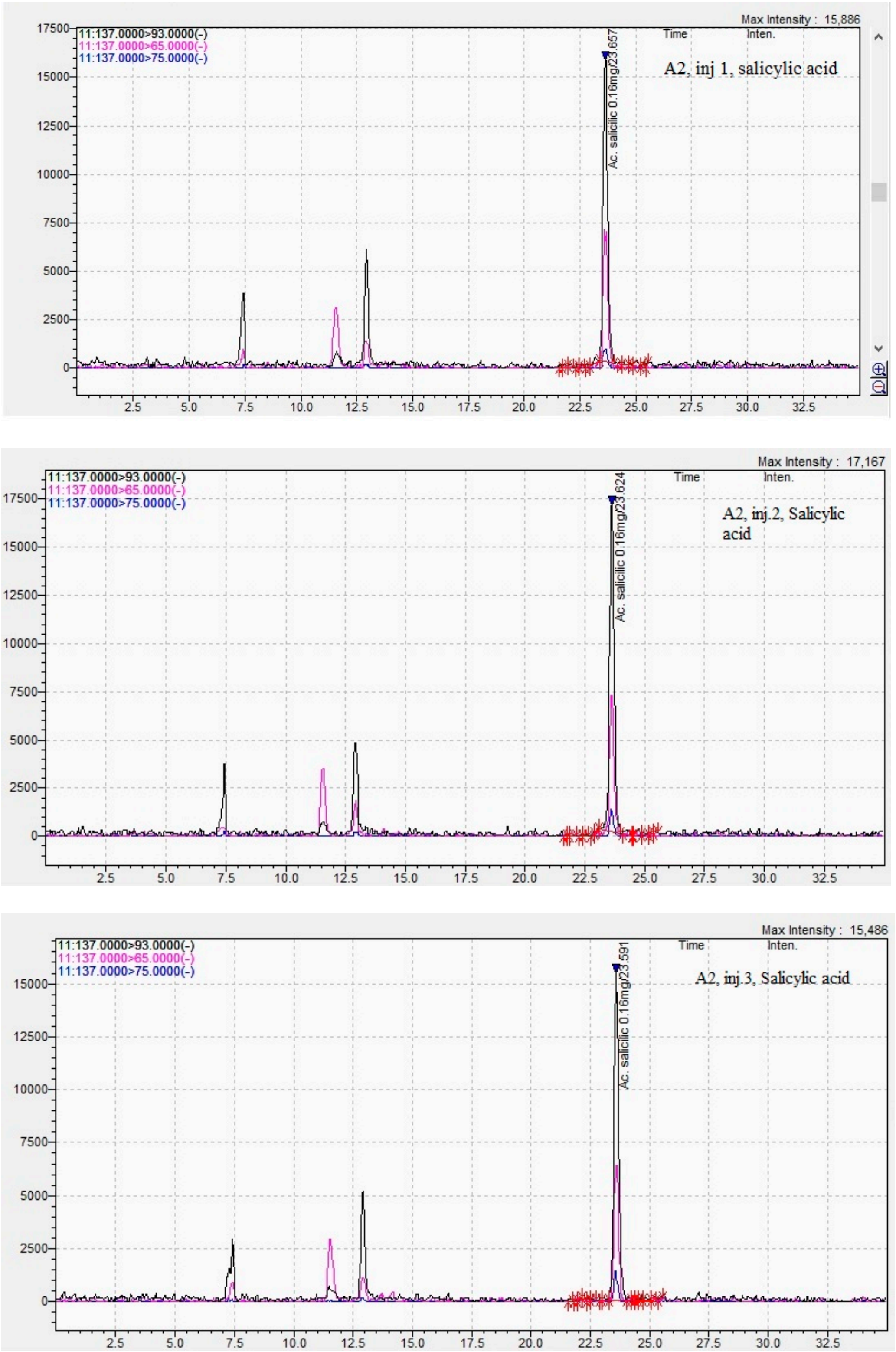

Figure S8. LC-MS chromatograms obtained for the identification of salicylic acid in the A2 sample (3 injections).

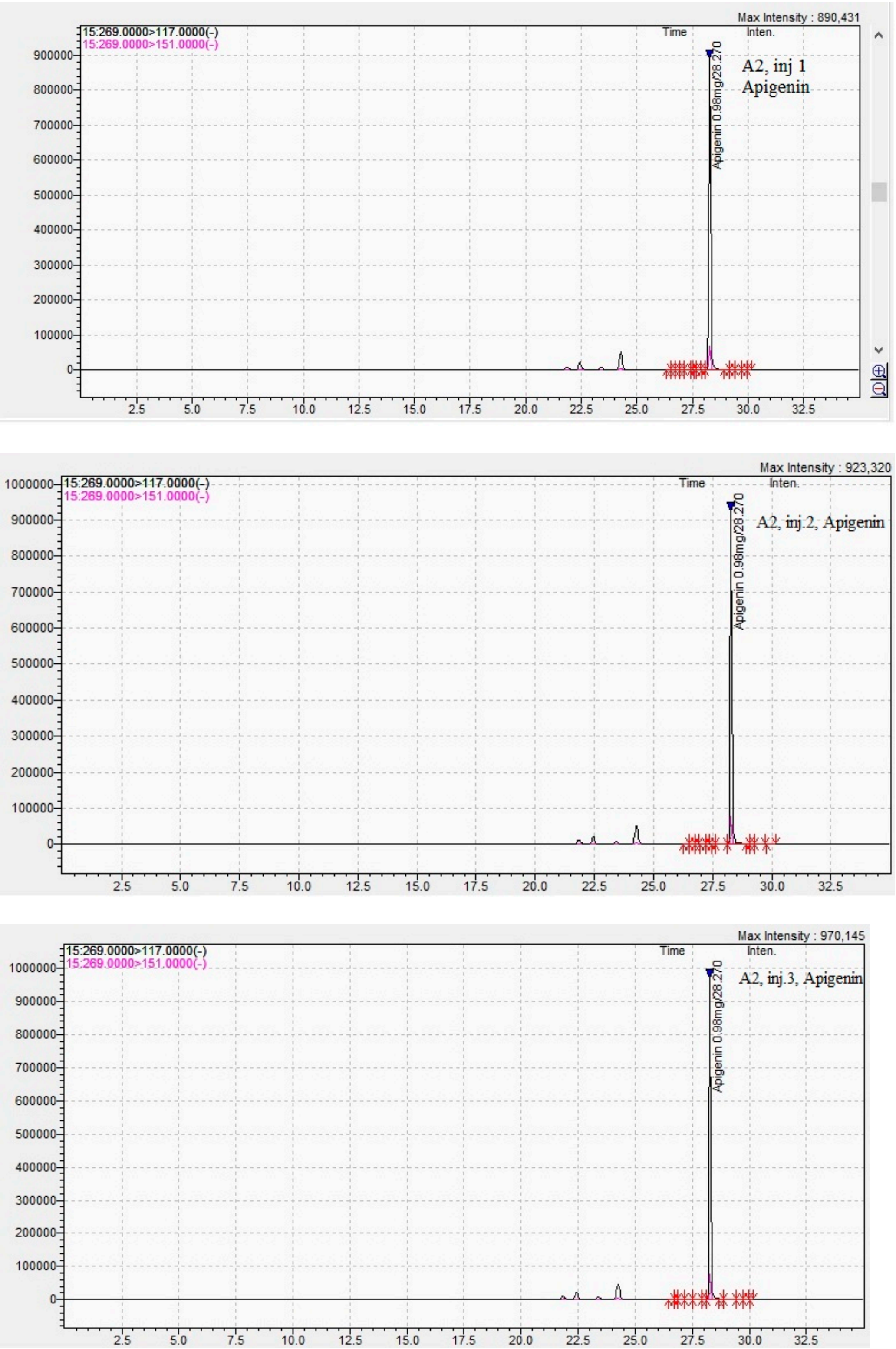

Figure S9. LC-MS chromatograms obtained for the identification of apigenin in the A2 sample (3 injections).

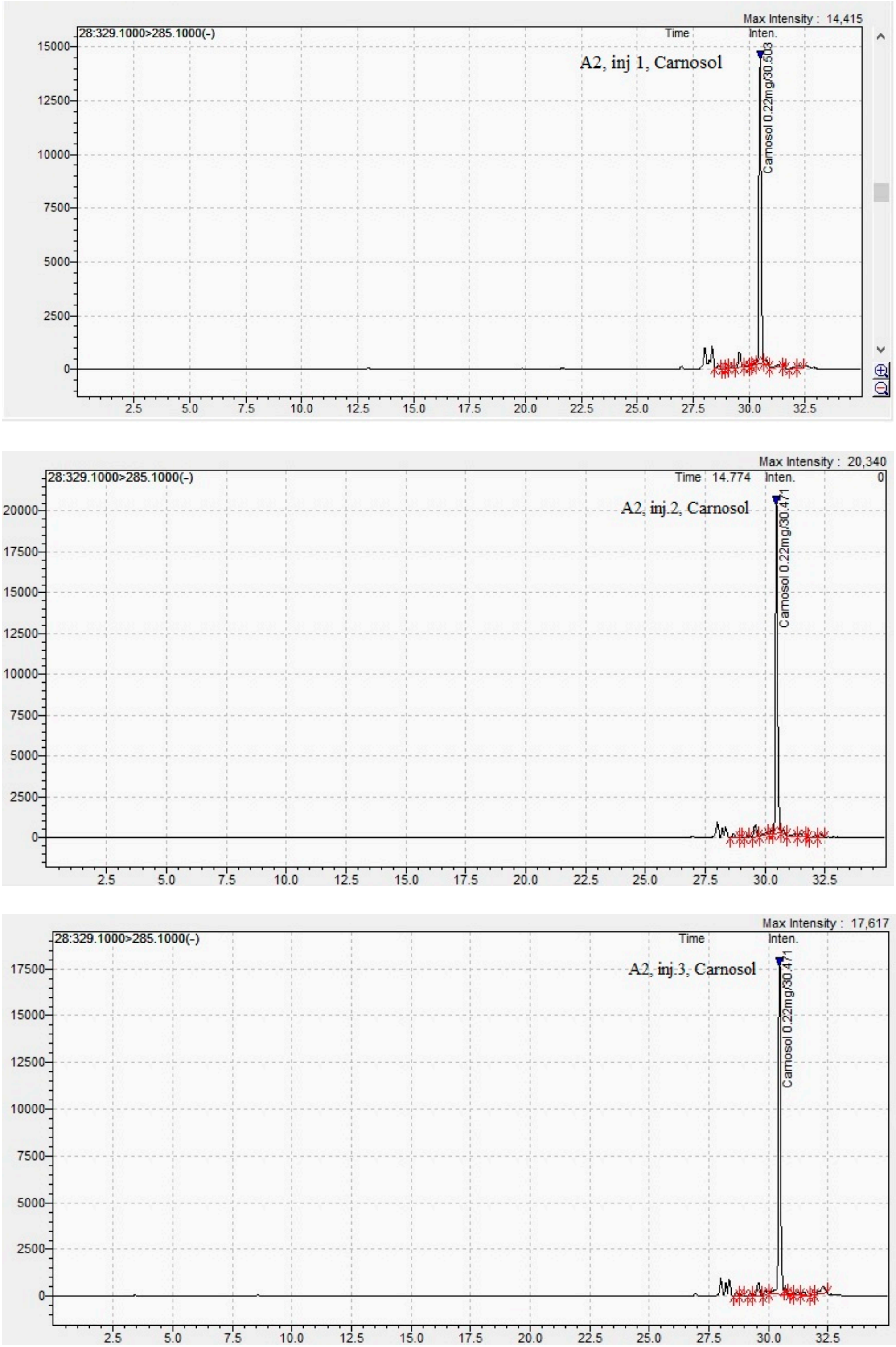

Figure S10. LC-MS chromatograms obtained for the identification of carnosol in the A2 sample (3 injections).

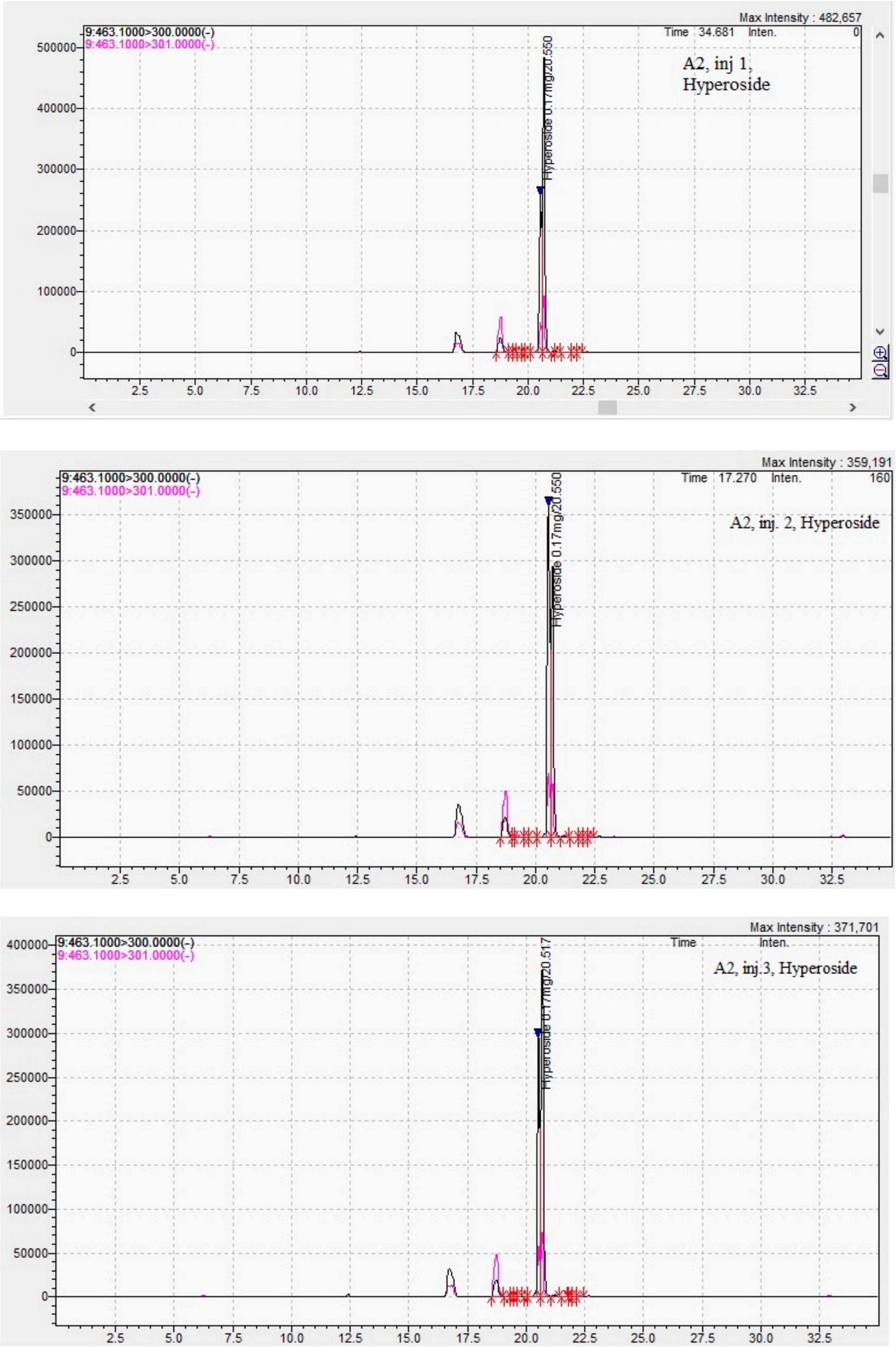

Figure S11. LC-MS chromatograms obtained for the identification of hyperoside in the A2 sample (3 injections).

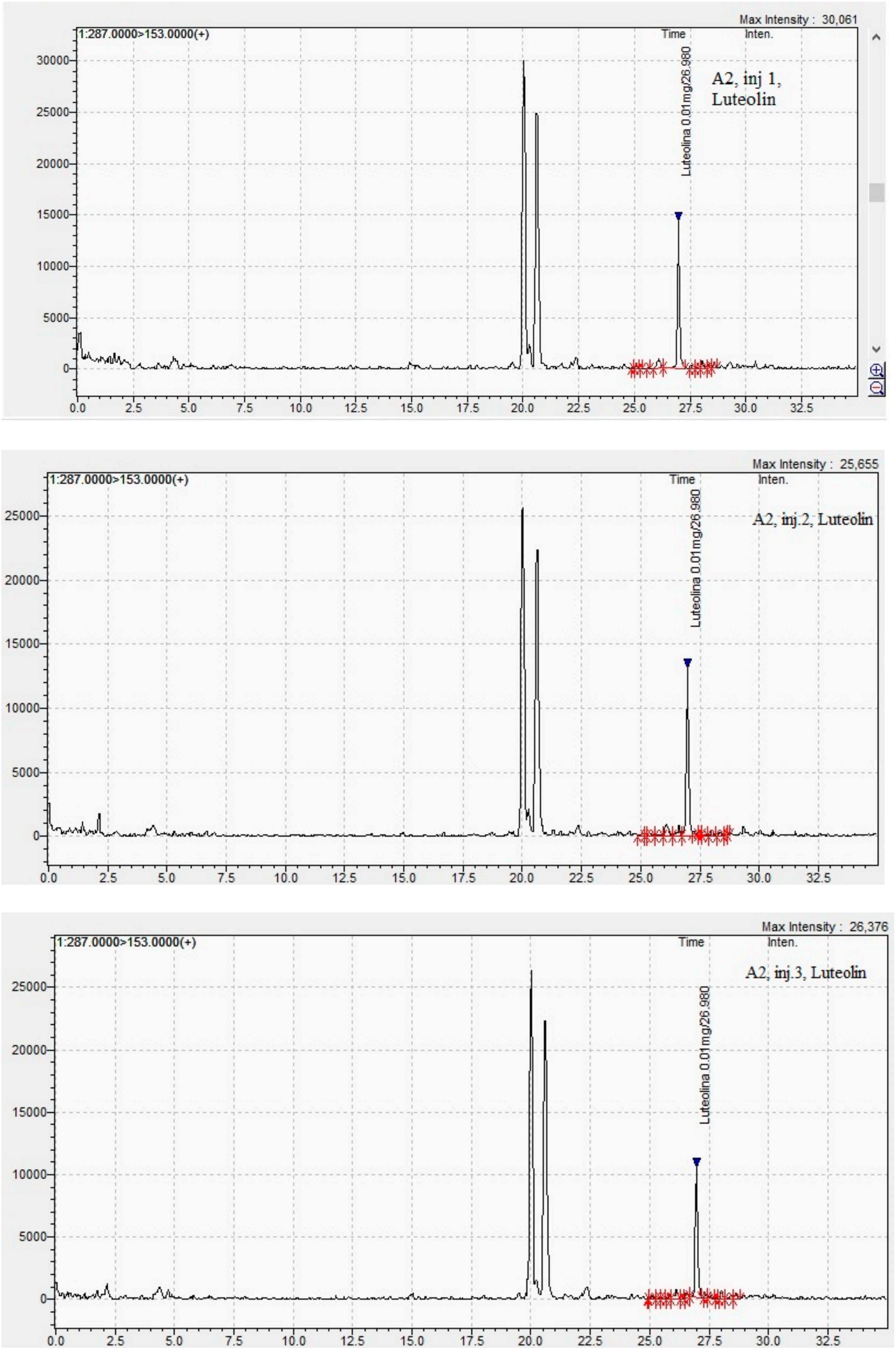

Figure S12. LC-MS chromatograms obtained for the identification of luteolin in the A2 sample (3 injections).

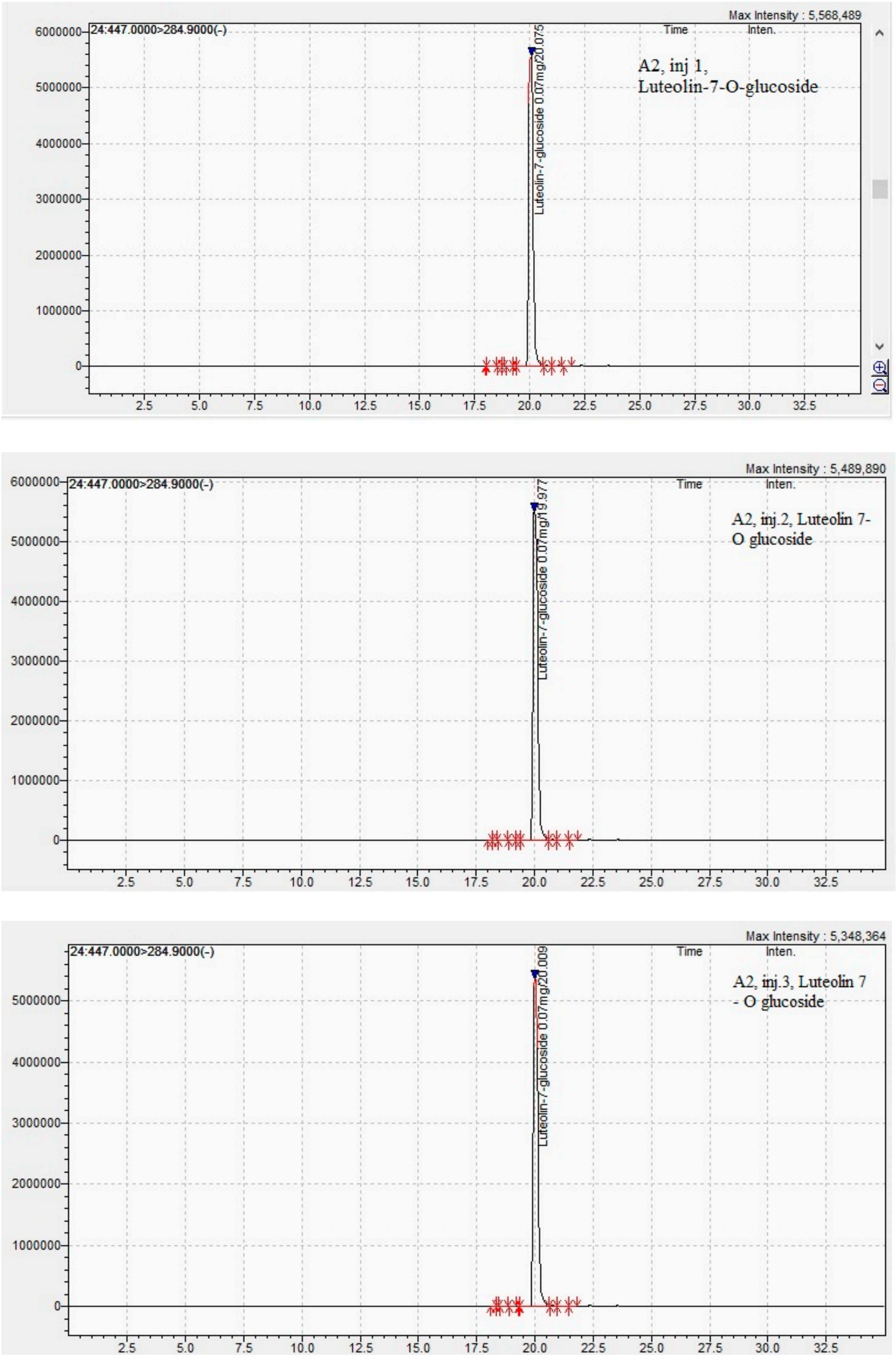

Figure S13. LC-MS chromatograms obtained for the identification of luteolin-7-O-glucoside in the A2 sample (3 injections).

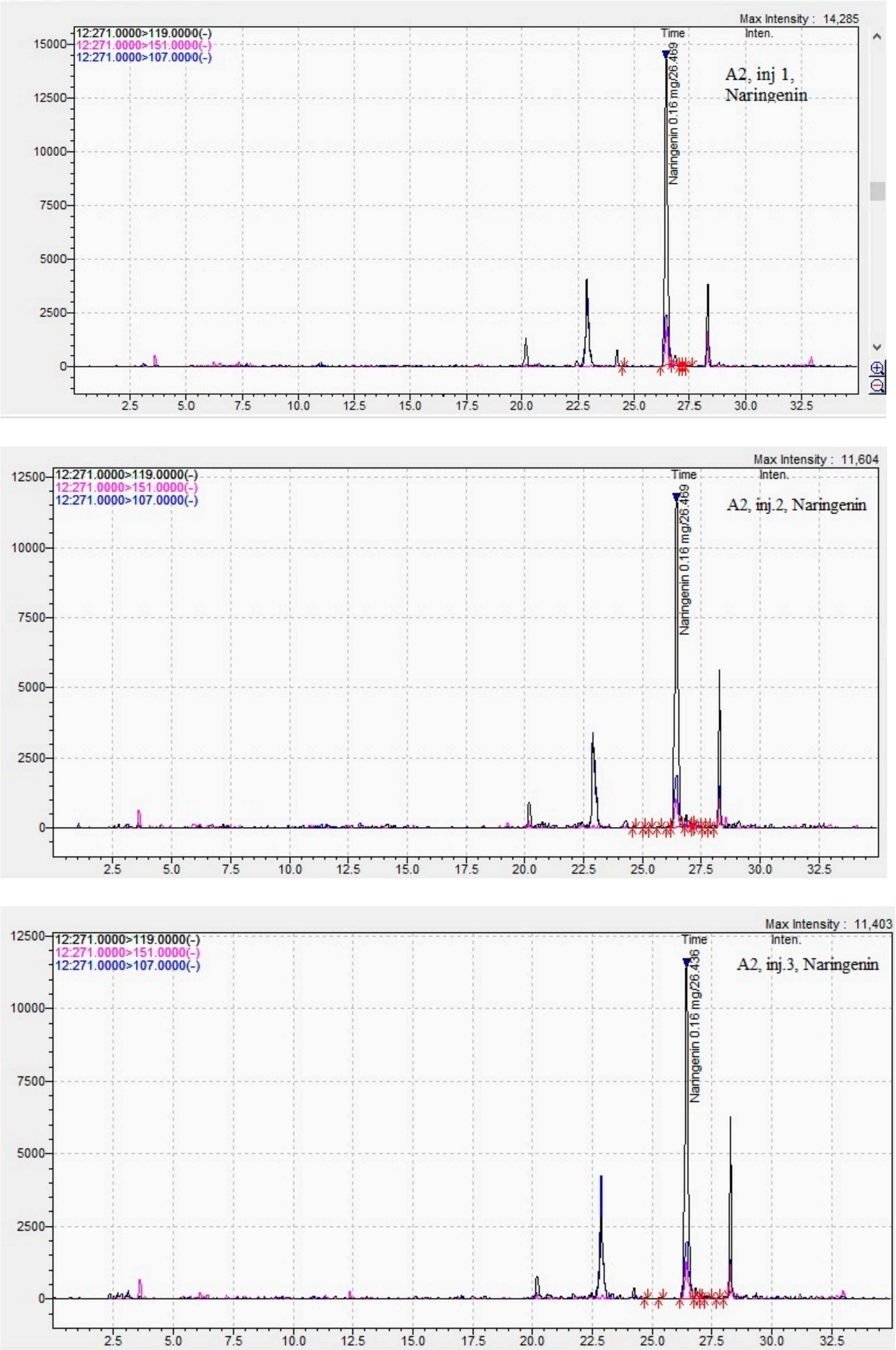

Figure S14. LC-MS chromatograms obtained for the identification of naringenin in the A2 sample (3 injections).

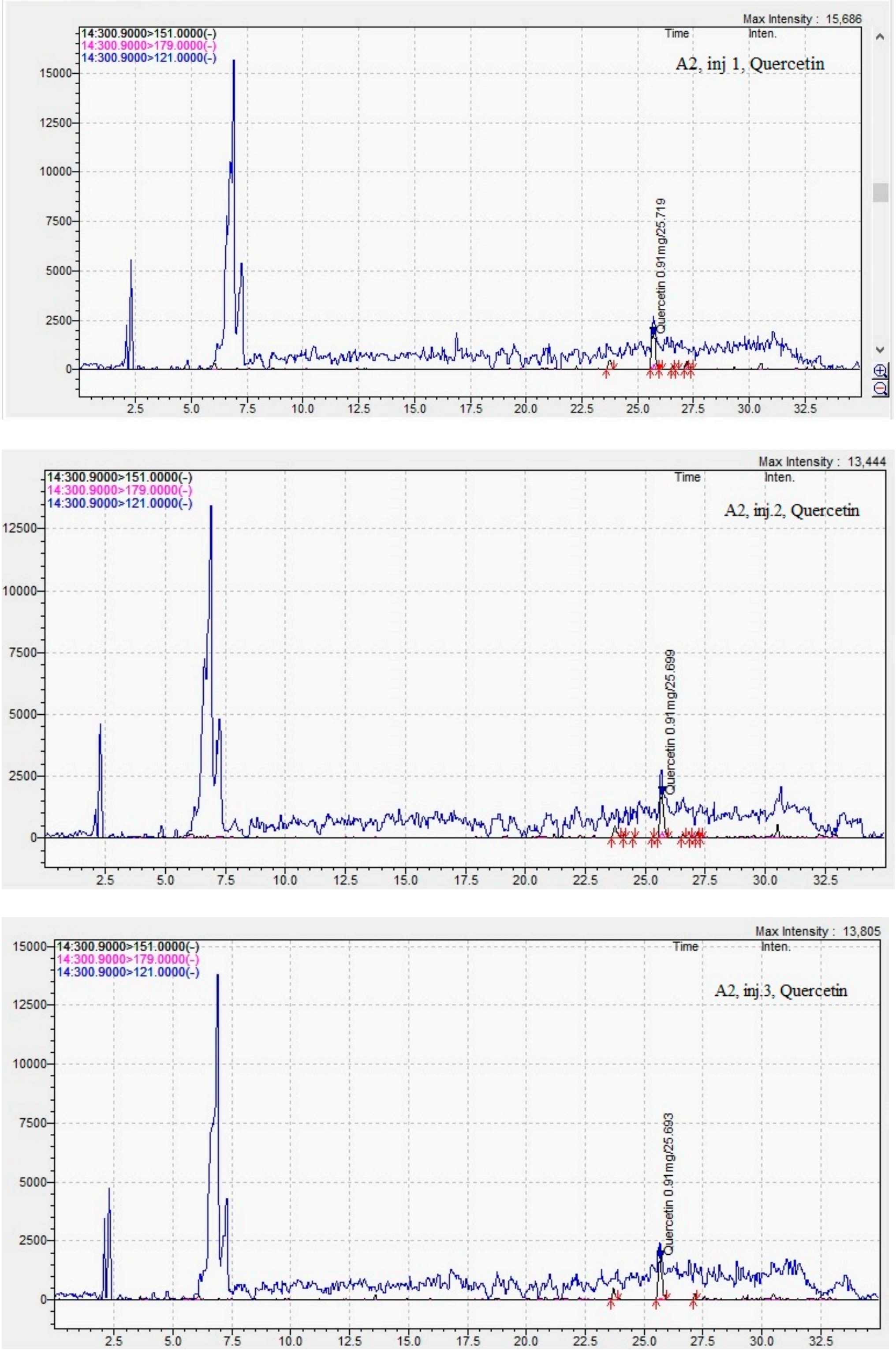

Figure S15. LC-MS chromatograms obtained for the identification of quercetin in the A2 sample (3 injections).

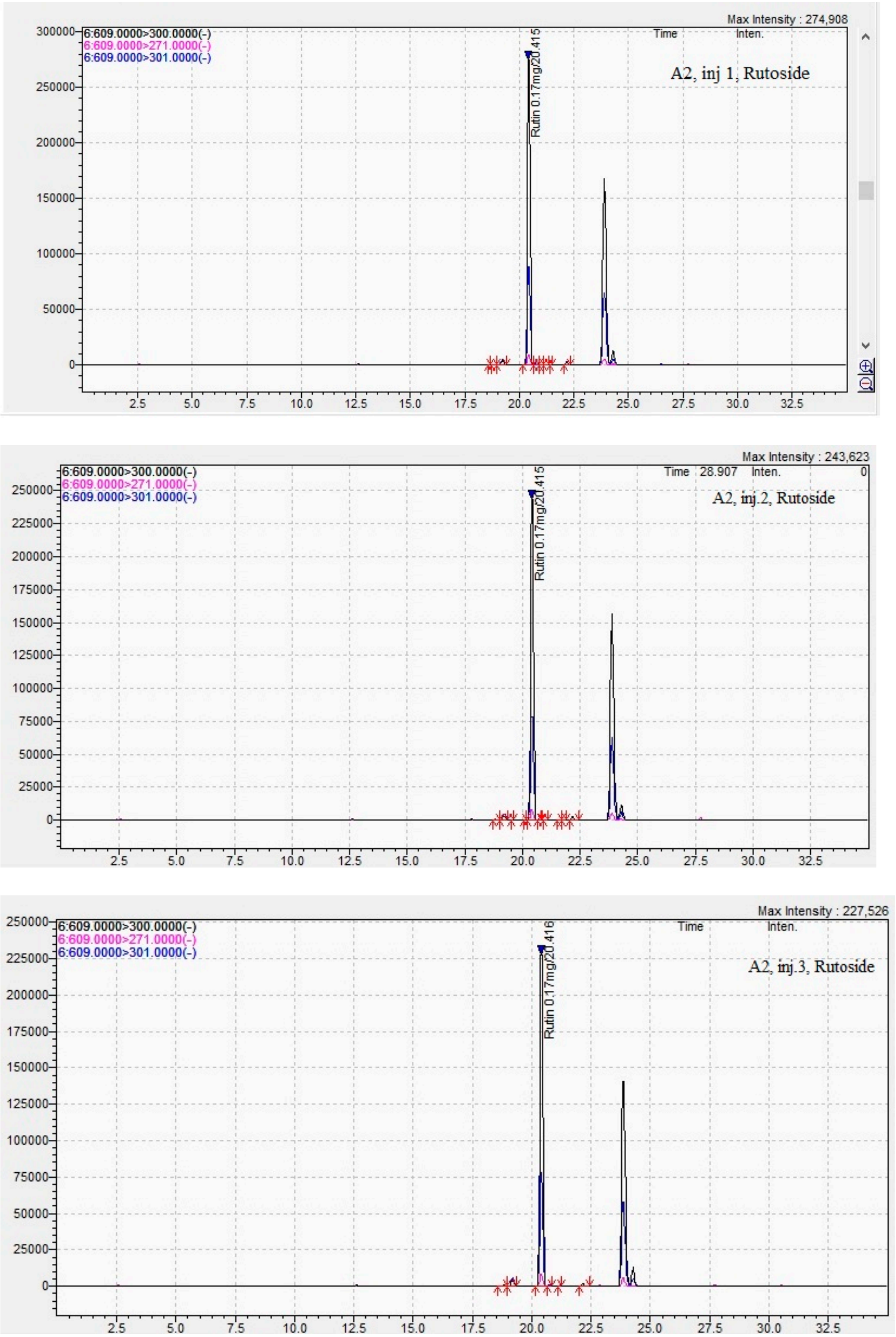

Figure S16. LC-MS chromatograms obtained for the identification of rutoside in the A2 sample (3 injections).

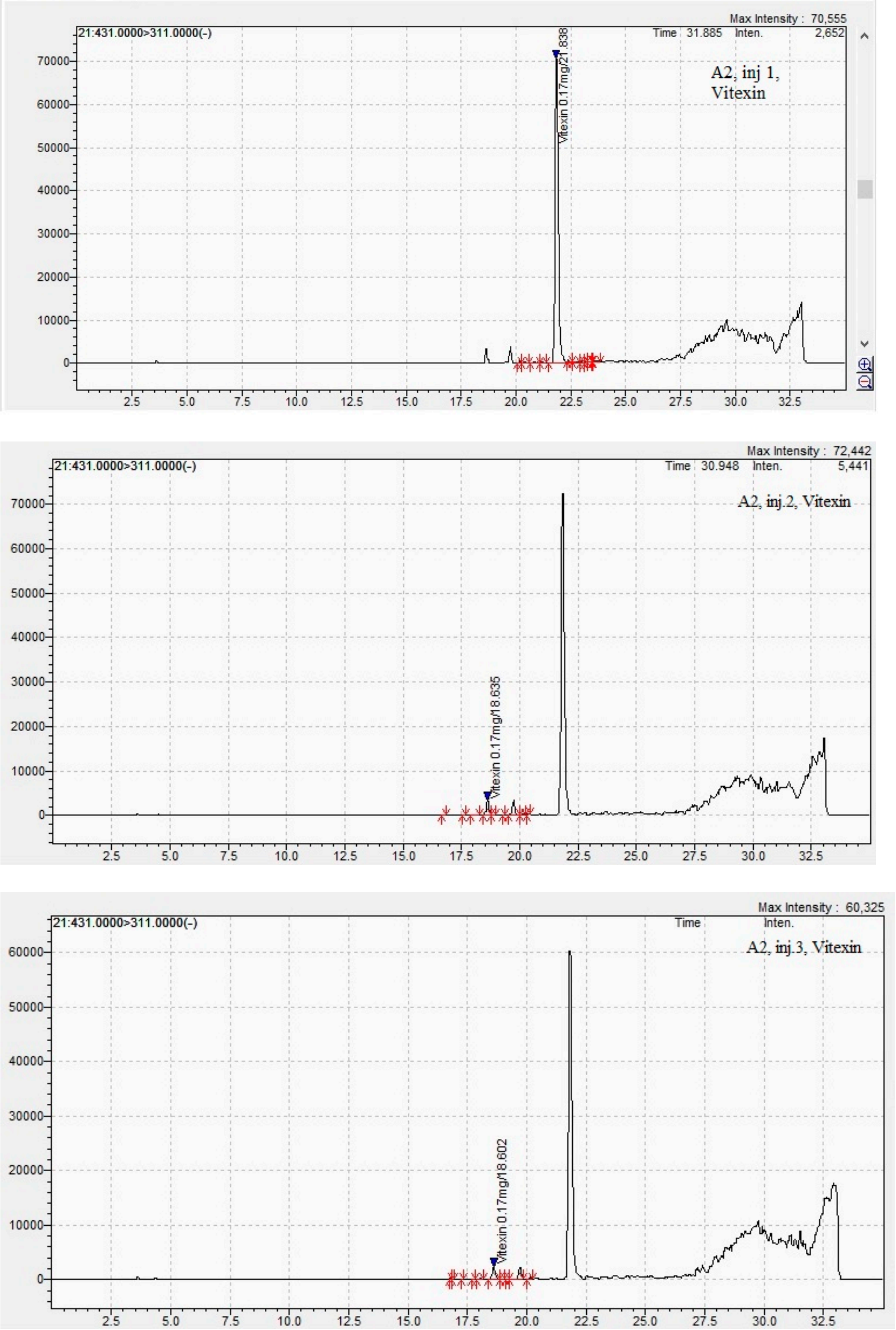

Figure S17. LC-MS chromatograms obtained for the identification of vitexin in the A2 sample (3 injections).

A2	A	Acontrol	I	I%	Conc (μg/mL)
1	0,916		0,057516	5,751621	6,25
2	0,813		0,163494	16,34942	12,5
3	0,7352		0,243544	24,35436	18,75
4	0,6314	0,9719	0,350345	35,03447	25
5	0,5465		0,437699	43,76994	31,25
6	0,4557		0,531125	53,11246	37,5
7	0,3679		0,621463	62,14631	43,75
8	0,2804		0,711493	71,1493	50

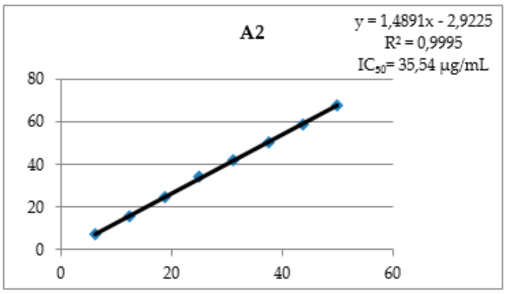

Figure S18. Determination of IC_{50} for the A2 sample. Note: I % = (A control − A sample/A control) × 100 where A control is the absorbance of the control, composed of the DPPH● radical solution + methanol (mixture containing all reagents except the sample) and A sample is the absorbance of DPPH radical + sample. The DPPH radical scavenging activity of the sample was expressed as IC_{50} (μg/mL), that is the concentration required to cause a 50% DPPH inhibition.

B2	A	Acontrol	I	I%	Conc (μg/mL)
1	1,1161		0,042714	4,271378	6,25
2	0,9737		0,164851	16,48512	12,5
3	0,8729		0,251308	25,1308	18,75
4	0,7782	1,1659	0,332533	33,25328	25
5	0,6667		0,428167	42,81671	31,25
6	0,5549		0,524059	52,40587	37,5
7	0,4253		0,635217	63,52174	43,75
8	0,3374		0,71061	71,06098	50

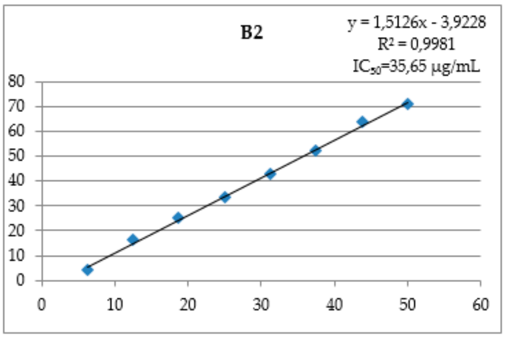

Figure S19. Determination of IC_{50} for the B2 sample. Note: I % = (A control − A sample/A control) × 100 where A control is the absorbance of the control, composed of the DPPH● radical solution + methanol (mixture containing all reagents except the sample) and A sample is the absorbance of DPPH radical + sample. The DPPH radical scavenging activity of the sample was expressed as IC_{50} (μg/mL), that is the concentration required to cause a 50% DPPH inhibition.

A1	A	Acontrol	I	I%	Conc (μg/mL)
1	0,9857		0,365048	36,50477	12,5
2	0,9045		0,417354	41,73538	25
3	0,8345		0,462445	46,24452	37,5
4	0,7246	1,5524	0,533239	53,32389	50
5	0,5742		0,630121	63,01211	62,5
6	0,4826		0,689127	68,91265	75
7	0,4012		0,741561	74,15615	87,5
8	0,3321		0,786073	78,60732	100

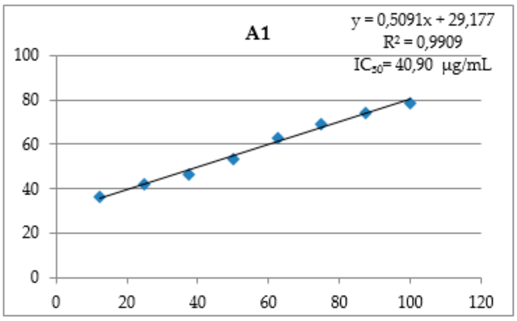

Figure S20. Determination of IC_{50} for the A1 sample. Note: I % = (A control − A sample/A control) × 100 where A control is the absorbance of the control, composed of the DPPH● radical solution + methanol (mixture containing all reagents except the sample) and A sample is the absorbance of DPPH radical + sample. The DPPH radical scavenging activity of the sample was expressed as IC_{50} (μg/mL), that is the concentration required to cause a 50% DPPH inhibition.

Ascorbic acid	A	Acontrol	I	I%	Conc (μg/mL)
1	1,2733		0,076851	7,685058	1,25
2	1,0846		0,213659	21,36591	2,5
3	0,9212		0,332125	33,2125	3,75
4	0,7812	1,3793	0,433626	43,36258	5
5	0,6262		0,546002	54,60016	6,25
6	0,4488		0,674618	67,46176	7,5
7	0,305		0,778873	77,88733	8,75
8	0,1443		0,895382	89,53817	10

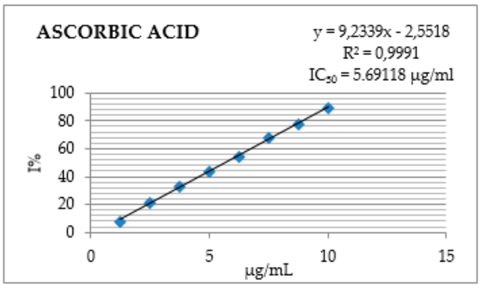

Figure S21. Determination of IC_{50} for the ascorbic acid. Note: I % = (A control − A sample/A control) × 100 where A control is the absorbance of the control, composed of the DPPH● radical solution + methanol (mixture containing all reagents except the sample) and A sample is the absorbance of DPPH radical + sample. The DPPH radical scavenging activity of the sample was expressed as IC_{50} (μg/mL), that is the concentration required to cause a 50% DPPH inhibition.

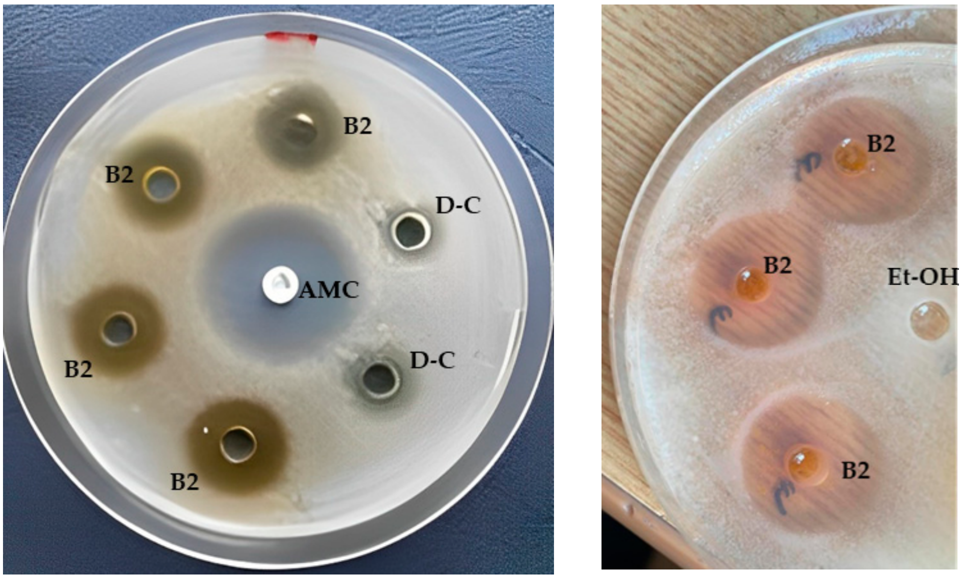

(a) (b)

Figure S22. *In vitro* antibacterial activity of the *D. moldavica* sample B2 by well diffusion method against reference strains: (**a**) *Staphylococcus aureus* ATCC 25923, (**b**) *Escherichia coli* ATCC 25922; positive (AMC, amoxicillin-clavulanic acid) and negative (70% Et-OH) control center placed; D-C (*D. moldavica* commercial product, data not included).

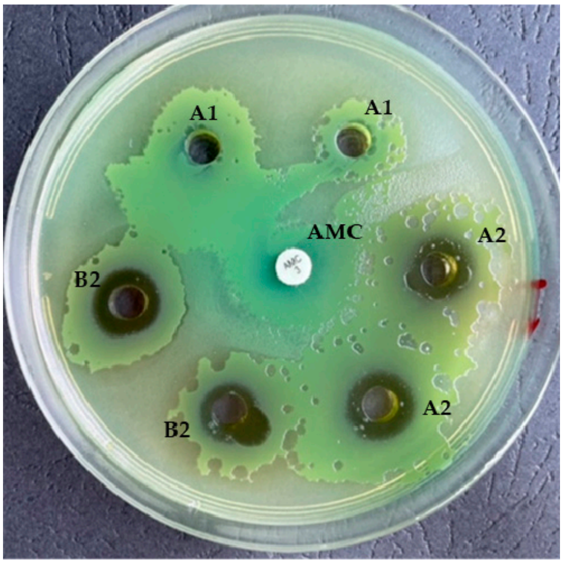

Figure S23. *In vitro* antibacterial activity of the *D. moldavica* samples (A1, A2, B2) as well as of the positive control (AMC, amoxicillin-clavulanic acid, center placed) by well diffusion method against *Pseudomonas aeruginosa* ATCC 27853, no inhibitory effect towards the reference strain.
